# Thy1 marks a distinct population of slow-cycling stem cells in the mouse epidermis

**DOI:** 10.1038/s41467-022-31629-1

**Published:** 2022-08-08

**Authors:** Elle Koren, Alona Feldman, Marianna Yusupova, Avihay Kadosh, Egor Sedov, Roi Ankawa, Yahav Yosefzon, Waseem Nasser, Stefanie Gerstberger, Liam B. Kimel, Noa Priselac, Samara Brown, Sam Sharma, Travis Gorenc, Ruby Shalom-Feuerstein, Hermann Steller, Tom Shemesh, Yaron Fuchs

**Affiliations:** 1 Laboratory of Stem Cell Biology and Regenerative Medicine, Department of Biology, Technion Israel Institute of Technology, Haifa, Israel; 2 Lorry Lokey Interdisciplinary Center for Life Sciences & Engineering, Technion Israel Institute of Technology, Haifa, Israel; 3 Laboratory of Biophysics, Department of Biology, Technion Israel Institute of Technology, Haifa, Israel; 4 Department of Genetics and Developmental Biology, The Rappaport Faculty of Medicine and Research Institute, Technion Israel Institute of Technology, Haifa, Israel; 5 Massachusetts General Hospital, Boston, CA USA; 6 Strang Laboratory of Apoptosis and Cancer Biology, The Rockefeller University, New York, New York, 10065 USA; 7 Present Address: Institute for Stem Cell Biology and Regenerative Medicine, Stanford University School of Medicine, Palo Alto, CA USA

**Keywords:** Regeneration, Skin stem cells, Self-renewal

## Abstract

The presence of distinct stem cells that maintain the interfollicular epidermis is highly debated. Here, we report a population of keratinocytes, marked by Thy1, in the basal layer of the interfollicular epidermis. We find that epidermal cells expressing differential levels of Thy1 display distinct transcriptional signatures. Thy1^+^ keratinocytes do not express T cell markers, express a unique transcriptional profile, cycle significantly slower than basal epidermal progenitors and display significant expansion potential in vitro. Multicolor lineage tracing analyses and mathematical modeling reveal that Thy1^+^ basal keratinocytes do not compete neutrally alike interfollicular progenitors and contribute long-term to both epidermal replenishment and wound repair. Importantly, ablation of Thy1^+^ cells strongly impairs these processes, thus indicating the non-redundant function of Thy1^+^ stem cells in the epidermis. Collectively, these results reveal a distinct stem cell population that plays a critical role in epidermal homeostasis and repair.

## Abstract (web summary)

Koren and Feldman et al. report a distinct population of Thy1-expressing stem cells in the basal layer of the mouse epidermis. These stem cells do not compete neutrally and contribute long-term to both epidermal replenishment and wound repair.

## Introduction

The interfollicular epidermis (IFE) is a heterogeneous tissue that undergoes continuous and constant replenishment throughout adulthood^1–3^. These features, akin to other highly regenerative epithelia such as the hair follicle (HF) and intestinal lining^4–6^, suggest the existence of a dedicated epidermal stem cell (SC) pool that maintains homeostatic regeneration and wound repair.

Presently, two models have been proposed for the maintenance of the IFE. One model suggests that the IFE is maintained by a homogenous population of committed progenitors/stem cells that stochastically balance differentiation and renewal^7,8^. Additional studies indicate that, in different skin regions, cells of the basal layer are heterogeneous in nature by housing a distinct slow-cycling epidermal SC population, which coexists with transient amplifying progenitors^9–18^. While both of these cell types express keratin-14, they can be separated based on transcriptional signature, rate of division, survival, and response to wounding^12,14,15,17,19^. Nonetheless, the existence of epidermal SCs remains controversial^7–9,13–15,17–26^.

Here, we report the identification of a distinct epidermal SC population, marked by the expression of Thy1, which plays a key role in IFE homeostasis and repair. Thy1^+^ epidermal SCs reside in the basal layer of the mouse dorsal skin, are distinct from T cells, cycle significantly slower than epidermal progenitors, express a unique gene signature, and can be easily expanded for extended periods. Furthermore, lineage-tracing and ablation experiments reveal that Thy1^+^ SCs regulate IFE replenishment, epidermal repopulation, and scar maintenance post wounding, indicating their important role in epidermal homeostasis and tissue regeneration.

## Results

### Thy1 is expressed in basal epidermal cells

**Fig. 1:**
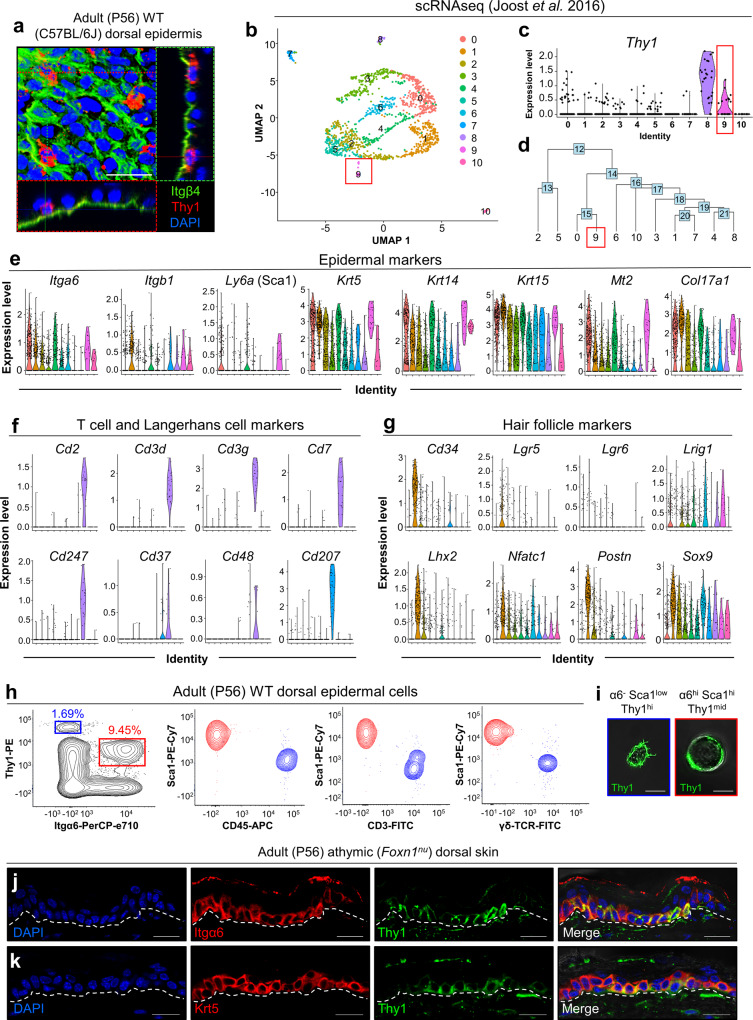
Thy1 marks a distinct subpopulation of basal epidermal cells. **a** Confocal image and 3D projections showing sagittal and transverse planes of wild-type adult (8-week-old) dorsal epidermis, immunostained against Thy1 and basement membrane marker Integrin-β4 (Itgβ4; CD104) [*n* *=* 3 mice]. **b** Clusters visualized via uniform manifold approximation and projection (UMAP). Adapted from scRNA-seq data of Joost et al., 2016^27^. **c**, **d** Resolution of two spatially distinct Thy1-expressing regions in the dataset. **e**–**g** Violin plots showing expression level distributions of (**e**) epidermal markers, (**f**) T cell/Langerhans cell markers, and (**g**) hair follicle markers. **h** Flow cytometry plots of adult (8-week-old) WT (C57BL/6 J) dorsal keratinocytes gated for Integrin-α6 (α6) and Thy1 intensity (α6^−^Thy1^+^; blue and α6^+^Thy1^+^; red), shown on Thy1 vs. Sca1. Cell populations gated for α6^hi^Thy1^+^ (red) and α6^−^ Thy1^+^ (blue) are shown on Sca1 vs. CD45, CD3 or γδ-TCR [*n* = 3 pooled mice]. (**i**) Representative confocal images of sorted α6^−^Sca1^low^Thy1^+^ and α6^hi^Sca1^hi^Thy1^+^ cells [*n* = 10,000 cells]. **j**, **k** Athymic nude mice (8-week-old; denoted *Foxn1*^*nu*^) dorsal skin immunostained for Thy1 and (**j**) Integrin-α6 (Itgα6) and (**k**) keratin-5 (Krt5) [*n* = 3 mice]. Dashed white lines demarcate the epidermis/dermis boundary. Scale bars: 100 μm (**a**), 20 μm (**j**, **k**), 10 μm (**i**).

Performing an unbiased analysis of a published single-cell RNA-seq (scRNA-seq) dataset of telogenic IFE cells^27^, we found that the *Thy1.2* gene is transcribed in basal keratinocytes. Thy1, also known as CD90, is a GPI-linked glycoprotein that has been described as a marker of stem cells (SCs) in various tissues^28–30^. To evaluate a potential involvement of Thy1^+^ cells in the adult interfollicular epidermis (IFE), we first examined distinct regions reported to house label-retaining cells in the mouse tail skin, where SCs are presumed to reside^15,25^. Utilizing adult 8-week-old (P56) tail skin of wild-type (WT; C57BL/6J) mice, we performed co-immunofluorescence using antibodies against Thy1 and the basement membrane marker Integrin-β4 (Itgβ4/CD104). We could detect Thy1^+^ cells in the label-retaining zones of the tail skin (Supplementary Fig. 1a)^15,25^. To confirm this observation, we exposed the non-label-retaining zones, which are normally obscured by hair follicles (HFs), and found no Thy1^+^ cells (Supplementary Fig. 1b). We further determined that Thy1^+^ cells were located in the basal layer of adult (8-week-old; P56) telogenic dorsal skins (Fig. 1a).

The expression of Thy1 in the epidermis, along with the expression of a CD3/V_γ_3/V_δ_1 TCR complex, has been characterized as a defining feature of dendritic epidermal T cells (DETCs)^31–35^. In the developing mouse embryo, V_γ_3/V_δ_1 TCR/Thy1^+^ T cells arise in the fetal thymus before homing to the skin between embryonic days 16 and 18 (e16-18)^36^. Thus, as a potential indication of non-T cell identity, we performed immunostaining on e15 embryos and could detect abundant levels of Thy1 protein throughout the basal layer of the developing dorsal epidermis (Supplementary Fig. 1c).

In order to unbiasedly determine whether epidermal Thy1^+^ cells could potentially encompass a non-T cell population, we analyzed the scRNA-seq dataset of IFE cells from Joost et al^27^. Unsupervised clustering was performed to identify unique cell clusters and visualized via uniform manifold approximation and projection (UMAP) (Fig. 1b). Cell clusters were annotated using a similar marker-based approach by Kasper and colleagues to identify distinct populations (Supplementary Fig. 1d)^27^. As reported, Thy1 expression coupled with the established T cell gene, *Cd3g*, resolved a T cell cluster (cluster 8; Fig. 1b–d and Supplementary Fig. 1d). However, we identified an additional cluster in the dataset with lower Thy1 expression throughout the cell population (cluster 9; Fig. 1b, c). Hierarchical clustering revealed these clusters to be relatively distant from one another, supporting the presence of a distinct cellular origin (Fig. 1d). As an important indication of non-T cell identity, this Thy1-expressing population displayed higher expression levels of basal epidermal markers *Krt14, Ly6a* (Sca1), and *Itga6* (Fig. 1e). Other highly expressed genes included *Mt2*, a basal IFE marker^27,37^, as well as the basal keratins *Krt5* and *Krt15* (Fig. 1e)^38,39^. Furthermore, cluster 9 distinctly expressed high levels of *Col17a1*, suggestive of its SC identity (Fig. 1e)^9–18^. Importantly, cluster 9 did not express known markers of basal T cells^27^ (Fig. 1f) and was negative for a panel of follicular and interfollicular SC markers, including *Cd34, Lgr5, Lgr6, Axin2, Lhx2, Dlx1, Slc1a3* and *Tbx1* (Fig. 1g and Supplementary Fig. 1e)^13,15,16,40–46^. The recently described epidermal markers *Klf5* and *Wnt4*^47,48^, as well as the keratinocyte differentiation markers *Lor* and *Flg2* were also not distinctively expressed in cluster 9^27^ (Supplementary Fig. 1e, f). We further determined that cluster 9 was negative for sebaceous gland and melanocyte markers (Supplementary Fig. 1g, h)^27,49–52^.

These data strongly indicate that Thy1 marks T cells as well as a distinct epidermal keratinocyte population in the mouse skin.

To characterize the Thy1^+^ cell population further, we processed whole adult telogenic (8-week-old) dorsal epidermis from WT mice for flow cytometry utilizing basal keratinocyte markers Integrin-α6 (Itgα6/CD49f) and Stem cell antigen 1 (Sca1)^53^. We could detect two distinct populations that could be separated based on Thy1 expression levels in complement with additional markers (Fig. 1h). Flow cytometry indicated that the Thy1 highest-expressing population was negative for Itgα6 and expressed low levels of Sca1 (denoted α6^−^Sca1^low^Thy1^+^). These findings suggest that these cells are not keratinocytes (Fig. 1h). However, the other distinct Thy1^+^ population, expressing moderately lower levels of Thy1, exhibited high intensities of both Itgα6 and Sca1 (denoted α6^hi^Sca1^hi^Thy1^+^). We determined that α6^hi^Sca1^hi^Thy1^+^ cells comprised approximately 10% of the total α6^+^Sca1^+^ IFE cells (Fig. 1h).

Serving as further validation, we employed additional antibodies for flow cytometry, including anti-CD45 (a pan immune cell marker), anti-CD3, and anti-γδ-TCR, and examined their expression in Thy1^+^ cells. These analyses confirmed that Thy1^+^ keratinocytes were indeed negative for DETC markers (Fig. 1h). Fluorescent-activated cell sorting (FACS) followed by high-resolution confocal microscopy further indicated that α6^hi^Sca1^hi^Thy1^+^ keratinocytes were larger and round, while Thy1^+^ non-keratinocytes were small and displayed highly dendritic morphology as expected of a homeostatic T cell (Fig. 1i).

Additional proof that Thy1 marks a distinct non-T-cell population of epidermal cells was obtained by performing immunofluorescence staining on dorsal skins from adult telogenic (P56) athymic (*Foxn1*^*nu*^) nude mice. Our data clearly demonstrate the presence of Thy1^+^ basal keratinocytes co-labeled with Itgα6 and Krt5 in this setting (Fig. 1j, k).

Taken together, these findings strongly indicate that Thy1 marks a distinct population of epidermal keratinocytes.

### Epidermal Thy1^+^ cells display SC characteristics

**Fig. 2:**
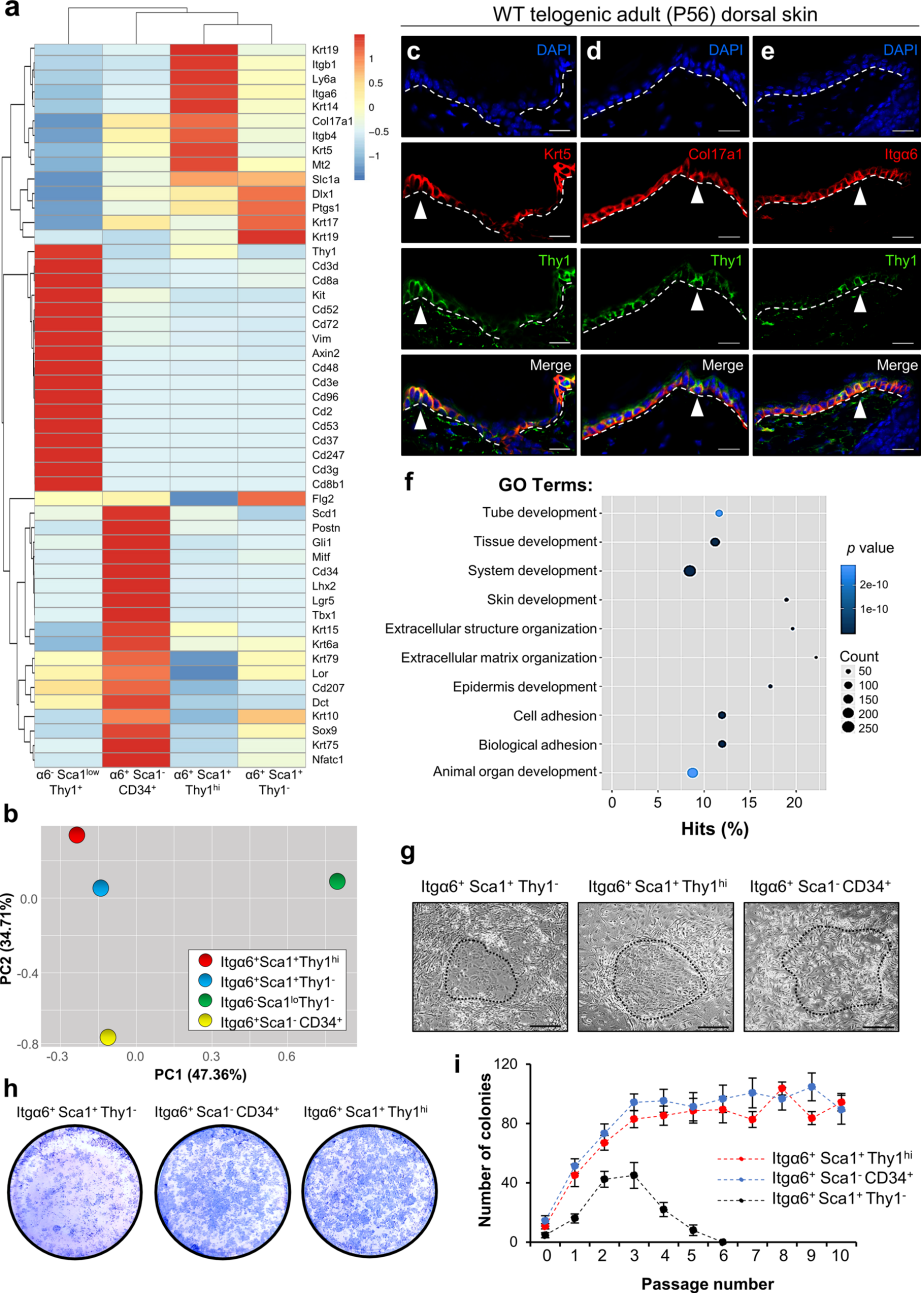
Thy1^+^ basal epidermal keratinocytes display self-renewal capacity. **a** Heatmap of absolute gene expression (RPKM values) scaled by gene based on bulk RNA-seq data comparing sorted populations of α6^−^Sca1^low^Thy1^+^ dendritic epidermal T cells (DETCs) [*n* = 4 mice], α6^+^Sca1^−^CD34^+^ hair follicle stem cells (HFSCs) [*n* = 5 mice], α6^+^Sca1^+^Thy1^+^ keratinocytes [*n* = 4 mice] and α6^+^Sca1^+^Thy1^−^ keratinocytes [*n* = 4 mice]. **b** Principal component analysis (PCA) showing PC-1 and PC-2 from RNA-seq data of sorted populations [*n* = 5 pooled mice]. **c**–**e** Co-immunostaining of Thy1 with (**c**) keratin-5 (Krt5), (**d**) Col17a1 and (**e**) Integrin-α6 (Itgα6) in sections of WT telogenic (P56) dorsal skins [*n* = 5 mice]. White arrowheads indicate cells expressing high levels of Thy1. **f** Gene ontology (GO) analysis of α6^+^Sca1^+^Thy1^hi^ vs. α6^+^Sca1^+^Thy1^−^ populations. Statistical testing was performed using the Wallenius’ distribution. **g** Images of holoclones grown 3 weeks post-plating of sorted α6^+^Sca1^+^Thy1^−^, α6^+^Sca1^+^Thy1^hi^, and α6^+^Sca1^−^CD34^+^ cells. **h** Rhodanile Blue staining of α6^+^Sca1^+^Thy1^−^ keratinocytes, α6^+^Sca1^−^CD34^+^ HFSCs and α6^+^Sca1^+^Thy1^hi^ cultures at passage 4 [*n* = 3 individual cultures each]. **i** Quantification for mean number of colonies formed during passaging [*n* = 3 individual cultures each]. Error bars represent ±S.E.M. All source data are provided as a Source Data file. Dashed white lines demarcate the epidermis/dermis boundary. Scale bars: 50 μm (**c**–**e**, **g**).

We next sorted epidermal cells from adult (8-week-old) WT dorsal skins and performed bulk RNA-seq followed by Principal Component Analysis (PCA) to compare the transcriptomic profiles of α6^−^Sca1^low^Thy1^+^ (DETCs), α6^+^Sca1^−^CD34^+^ (hair follicle stem cells; HFSCs), and α6^+^Sca1^+^Thy1^hi^ and α6^+^Sca1^+^Thy1^−^ keratinocytes (Fig. 2a and Supplementary Fig. 2a–d). As expected, and consistent with the dataset by Joost et al., the IFE Sca1^+^ populations expressed relatively low levels of HFSC markers including *Cd34*,* Lgr5*, *Sox9*, *Gli1*, *Lhx2*, *Nfatc1*, and *Tbx1* in contrast to the α6^+^Sca1^−^CD34^+^ HFSC population^6,40–46^ (Fig. 2a). Additionally, α6^+^Sca1^+^Thy1^hi^ cells displayed either low or barely detectable levels of the reported IFE SC markers *Dlx1* and *Axin2*^13,15^, while levels of *Ly6a* (Sca1) and *Itga6*, *Itgb4*, and *Itgb1* were elevated^54,55^ (Fig. 2a). Transcript levels of basal *Krt5* and *Krt14* were also enhanced in α6^+^Sca1^+^Thy1^hi^ cells, further indicating that they represent a subpopulation of basal keratinocytes (Fig. 2a). In line with the Joost et al. dataset, we detected high expression of* Col17a1* in the α6^+^Sca1^+^Thy1^hi^ population (Fig. 2a). Of note, we verified that α6^+^Sca1^+^Thy1^hi^ cells did not display a T cell signature, in contrast to the α6^−^Sca1^low^Thy1^+^ population that was highly enriched for genes such as *Cd2, Cd3d, Cd3e, Cd3g, Cd8a and Cd96* (Fig. 2a). Likewise, the presence of major differences between these cell populations was observed through PCA (Fig. 2b).

In order to confirm the transcriptomic profile of the α6^+^Sca1^+^Thy1^hi^ population, we performed immunostaining of adult telogenic (P56) dorsal skins from WT mice. These experiments demonstrated co-localization of Thy1^+^ dorsal skin cells with the basal epidermal markers Krt5, Krt14, Itgα6, Itgβ4, and Col17a1 (Fig. 2c–e and Supplementary Fig. 2e–g). Of note, cells expressing the highest levels of Thy1 coincided with cells displaying higher levels of Krt5, Krt14, Itgα6, Itgβ4, and Col17a1 (Fig. 2c–e and Supplementary Fig. 2e–g).

We also asked whether Thy1^+^ cells exist in other skin regions of the mouse body. For these analyses, we harvested adult telogenic (P56) ventral skin, tail skin, and glabrous skin of the hind paw for immunostaining against Thy1 and Krt5. In all examined skin regions we detected the presence of Krt5^+^ Thy1^+^ cells (Supplementary Fig. 3a–c).

Of note, Gene Ontology (GO) analysis of differentially expressed genes in the α6^+^Sca1^+^Thy1^hi^ (vs. α6^+^Sca1^+^Thy1^−^) population revealed enrichment for terms including skin and epidermal development, thereby supporting a functional role in tissue maintenance (Fig. 2f).

In the classic hierarchical model, the epidermis is heterogeneously maintained by a slow-cycling population of SCs that self-renew and give rise to transit-amplifying progenitors^9–18^. To investigate whether Thy1^+^ keratinocytes display self-renewal capacity, we cultured both α6^+^Sca1^+^Thy1^+^ and α6^+^Sca1^+^Thy1^−^ cells isolated from adult telogenic (P56) WT dorsal epidermis. Additionally, we also cultured α6^+^Sca1^−^CD34^+^ HFSCs as an SC control. After eight weeks, we evaluated their colony-forming efficiency and found that α6^+^Sca1^+^Thy1^hi^ cells generated colonies at a comparable efficiency to α6^+^Sca1^−^CD34^+^ HFSCs (Fig. 2g). In contrast, α6^+^Sca1^+^Thy1^−^ cells exhibited a marked reduction in colony size (Fig. 2g). Furthermore, serial dilution and long-term passaging indicated that, unlike α6^+^Sca1^+^Thy1^−^ keratinocytes, which dwindled away after approximately five passages, α6^+^Sca1^+^Thy1^hi^ cells could be expanded over time (Fig. 2h, i). Similar expansion capability was also observed in α6^+^Sca1^−^CD34^+^ HFSCs (Fig. 2h, i). Taken together, our findings show that, in contrast to Thy1^−^ IFE cells that can amplify in a transient fashion, α6^+^Sca1^+^Thy1^hi^ cells can be expanded for extended periods of time and display higher clonogenic capacity; features suggestive of SC identity.

### Thy1^+^ SCs contribute to long-term epidermal replenishment

**Fig. 3:**
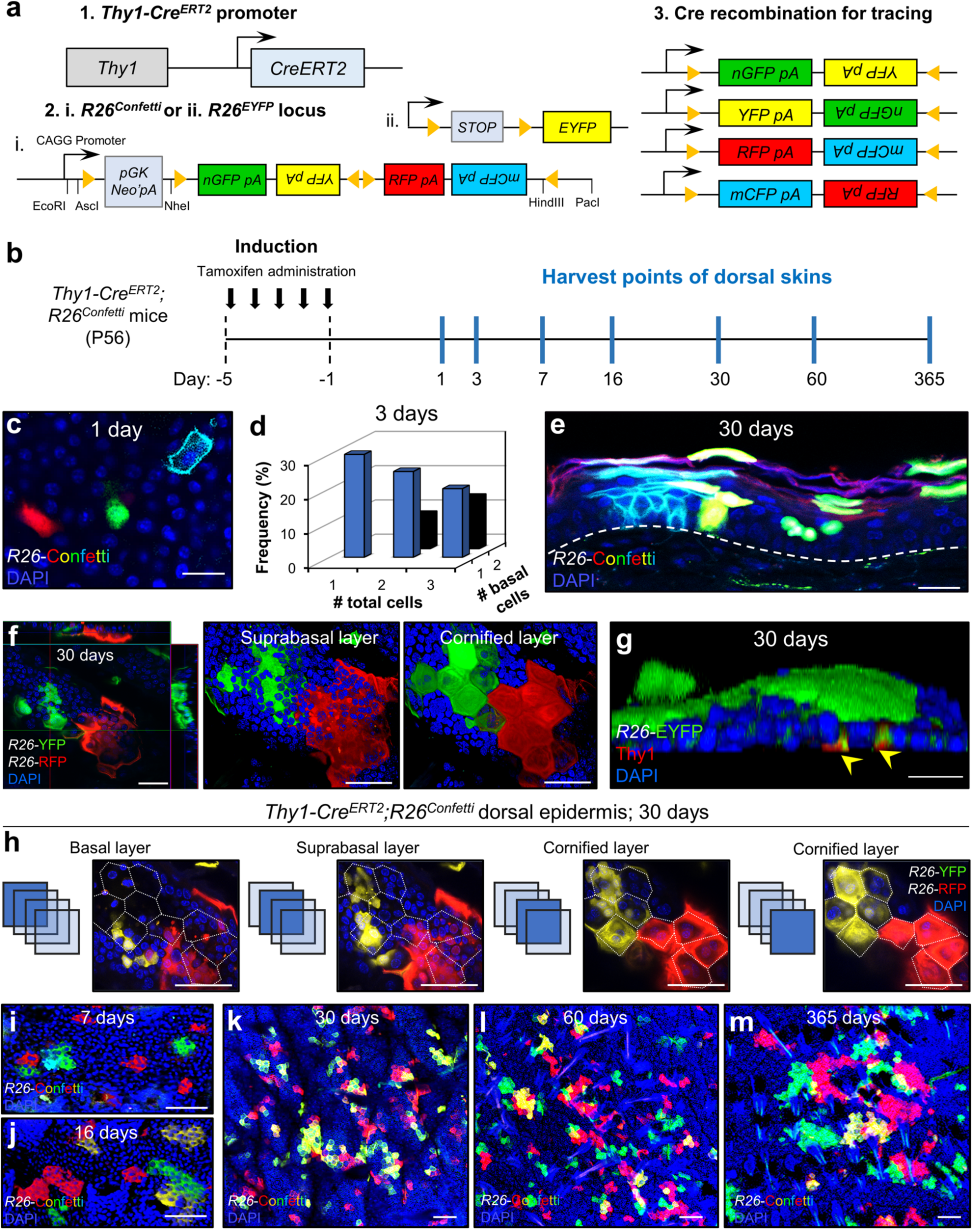
Thy1^+^ cells replenish the adult mouse dorsal epidermis over time. **a** Genetic strategy used to induce multicolor Confetti expression in Thy1^+^ cells. **b** Schematic of induction and harvesting regimen of the dorsal skin. **c** Image of Confetti-labeled cells in the basal layer after 1 day post induction [*n* = 5 mice]. **d** Frequency of clones comprised of more than one Thy1^+^ labeled cell at 3 days post induction [*n* = 90 clones from 4 pooled mice]. Source data are provided as a Source Data file. **e** Confocal image of sectioned epidermis at day 30 post-induction showing Confetti expression throughout the epidermal layers [*n* = 5 mice]. Dashed white line demarcates epidermis/dermis boundary. **f** Z-stack images of induced dorsal epidermis after 30 days showing formation of labeled cornified units [*n* = 5 mice]. **g** 3D rendered confocal image of *Thy1-Cre*^*ERT2*^;*R26*^*EYFP*^ dorsal skin at 30 days post induction co-stained with Thy1 (yellow arrows) [*n* = 5 mice]. **h** Z-stack sections of dorsal skin interfollicular epidermis (IFE) at 30 days post induction showing basal attachment. Dotted white lines marks polygonal projection of a cornified cell [*n* = 5 mice]. **i**–**m** Maximum projection confocal images of Thy1^+^ basal cell-derived clones at (**i**) 7 days, (**j**) 16 days, (**k**) 30 days, (**l**) 60 days, and (**m**) 365 days post induction [*n* = 4 mice per time point]. Scale bars: 100 μm (**k**–**m**), 50 μm (**e**, **f**, **h**–**j**), 20 μm (**c**, **g**).

A key characteristic of an SC is its capacity to differentiate into mature cells^1–3^. To examine the lineage potential of Thy1^+^ cells, we crossed inducible *Thy1-CreERT2* (Slick-H) mice. In these animals, back-to-back *Thy1* promoters were used in order to drive both CreERT2 and to specifically label neurons with EYFP. As expected, we detected high levels of EYFP in the brain, but not in epidermal cells (Supplementary Fig. 5a, b). We reasoned that although the EYFP was sub-detectable in the epidermis, limited expression of Cre in these mice would still mediate Cre recombination and thus could potentially be utilized for performing lineage tracing of Thy1^+^ epidermal cells. Therefore, we crossed the *Thy1-Cre*^*ERT2*^ (Slick-H) mice to either *R26*^*EYFP*^ or *R26*^*Confetti*^ mice^56,57^ (Fig. 3a).

To examine and verify the specificity of our lineage-tracing system we first investigated whether α6^+^Sca1^+^Thy1^+^ keratinocytes could be sufficiently induced. For this aim, we processed adult telogenic dorsal skins at 24 hours post-induction for flow cytometry and determined that α6^+^Sca1^+^Thy1^+^ keratinocytes become labeled with EYFP (Supplementary Fig. 4a). Of note, we could detect EYFP^+^ fluorescent cells largely in the Thy1^hi^ keratinocyte population. Surprisingly, we did not observe EYFP expression in Thy1^+^ DETCs (Supplementary Fig. 4a). We hypothesize that the reason for this may be because the Thy1-Cre^ERT2^ cassette is incorporated in a heterochromatic region or adjacent region that lacks the required cis-regulation in T cells^58^, but not in the Thy1^+^ SCs.

We further validated the expression of basal keratinocyte markers Sca1 and Itgα6, as well as EYFP and Thy1 using ImageStream analysis (Supplementary Fig. 4b). In addition, we performed bulk RNA-seq on sorted populations of both α6^+^Sca1^+^Thy1^hi^ and α6^+^Sca1^+^Thy1^hi^YFP^+^ cells, which indicated that labeled cells express epidermal SC markers after recombination (Supplementary Fig. 4c).

We next performed lineage tracing in the dorsal skin, starting from the adult telogen phase (P56) using either the inducible *Thy1-Cre*^*ERT2*^*;R26*^*EYFP*^ or *Thy1-Cre*^*ERT2*^*;R26*^*Confetti*^ mice (Fig. 3b). In the latter, upon Cre activation and recombination, Thy1^+^ cells randomly express one of four fluorescent proteins including nuclear GFP (nGFP), membranous CFP (mCFP), RFP, or YFP, which are localized to different cellular compartments^57^.

As an important first step, we calibrated our induction protocol to ensure single-cell labeling of Thy1^+^ cells (Fig. 3c, d and Supplementary Fig. 5c, d). We did not observe any induced cells or clonal events in the bulge, hair germ, dermal papilla or isthmus, although occasionally we could detect events in the infundibulum (IFD) and sebaceous gland (SG) base (Supplementary Fig. 5e). Strikingly, after 30 days, we observed clonal trails emanating from the basal layer to the upper differentiated layers, indicating the participation of the Thy1^+^ cell population in epidermal homeostasis (Fig. 3e, f). We next determined that initial clones displayed a marked Thy1^+^ cell and confirmed that the trace indeed originated from the basal layer (Fig. 3g, h). Moreover, the epidermis appeared as a mosaic of labeled clones where each individual clone displayed a polygonal shape (e.g., pentagon or hexagon), which was defined by the perimeter of the cornified layer (Fig. 3f, g). These structures appeared reminiscent of the units described by the epidermal proliferating unit (EPU) hypothesis, which predicts that each EPU harbors on average 10 cells in the basal layer with one slow-cycling SC positioned in its center^59^. Importantly, the EPU columns we identified did not directly extend from the basal to the cornified layer, since we could detect cells outside the polygonal projections (Fig. 3h). Specifically, although the majority of labeled basal cells aligned vertically into the borders of the EPU, in many instances the suprabasal cells appeared to cross the projected border, while cells of the granular layer were committed to the cornified layers above (Fig. 3h). These findings are consistent with reports that examined the spatiotemporal coordination in the mouse ear skin^8,19^.

At distinct time points including 7, 16, 30, 120, and 365 days post-induction we could clearly detect increasing amounts of EPU-like structures, demonstrating the long-term contribution of Thy1^+^ basal cells (Fig. 3i–m). Importantly, after 1-year post-induction we could detect EPU-like structures with basal attachment and confirmed that tracing events could only be seen in the IFE at this later time point (Supplementary Fig. 5f, g).

Taken together, these data indicate that Thy1 marks a distinct population of SCs that can differentiate and contribute to epidermal homeostasis over prolonged periods.

### Thy1^+^ basal SCs exhibit distinctive cycling activity

**Fig. 4:**
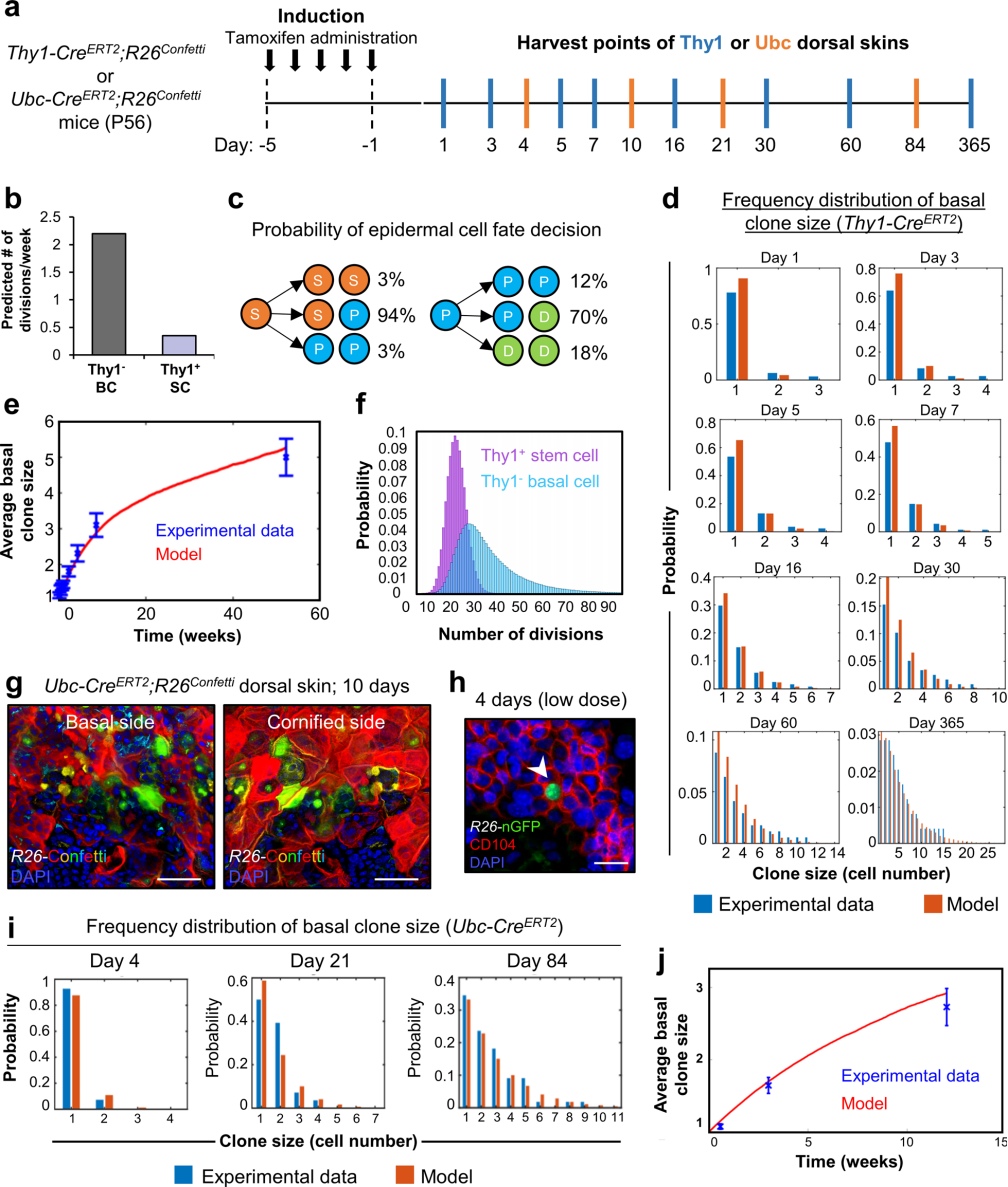
Thy1^+^ cells are a functionally distinct population of basal epidermal stem cells. **a** Schematic for induction and harvesting of Thy1 or Ubc dorsal skins. **b** Division rates of basal Thy1^+^ stem cells (SCs) and Thy1^−^ basal cells (BCs) determined from *Thy1-Cre*^*ERT2*^*;R26*^*Confetti*^ mice [*n* = 6 mice]. **c** Model probability for division symmetries of Thy1^+^ SCs and Thy1^−^ progenitor cells. **d** Basal clone size distributions at different time points following Thy1^+^ induction [*n* = 6 mice]. Blue columns represent the experimental data and red columns correspond to the model predictions. **e** Average basal clone size over time following Thy1^+^ induction [*n* = 6 mice]. Blue points represent the data taken from 1 to 365 days post induction. Red curve represents model prediction. **f** Model predictions for number of divisions of Thy1^+^ SCs and transit-amplifying cells at 365 days. (**g**) Maximum projection image of *Ubc-Cre*^*ERT2*^*;R26*^*Confetti*^ dorsal epidermis at 10 days post induction showing basal (left panel) and cornified (right panel) sides [*n* = 3 mice]. **h** Image of *Ubc-Cre*^*ERT2*^*;R26*^*Confetti*^ dorsal epidermis at 4 days post induction using low-dose tamoxifen [*n* = 3 mice]. Arrowhead indicates single induced nGFP^+^ basal cell. **i** Basal clone size distributions at different time points following random Ubc induction in *Ubc-Cre*^*ERT2*^*;R26*^*Confetti*^ mice [*n* = 6 mice]. Blue columns represent the experimental data and red columns correspond to the model predictions. **j** Average basal clone size over time following Ubc induction [*n* = 6 mice]. Blue points represent the data at 4, 21, and 84 days post induction. The red curve represents model prediction. All error bars show ±S.E.M. All source data are provided as a Source Data file. Scale bars: 50 μm (**g**), 10 μm (**h**).

We next sought to understand whether Thy1^+^ keratinocytes qualify as a slower-cycling SC population^14^ or compete neutrally like the majority of IFE progenitors^7,8^. For this aim, we performed detailed clonal analyses and mathematical modeling of the expansion dynamics of Thy1^+^ and Thy1^−^ basal clones over time in traced *Thy1-Cre*^*ERT2*^*;R26*^*Confetti*^ mice (Fig. 4a). Specifically, the clone size distribution of Thy1^+^ cells over time indicated 0.35 divisions/week; a rate considerably slower than 2.2 divisions/week of Thy1^−^ basal cells (BCs; Fig. 4b). Our estimation of the cellular turnover in the IFE is comparable with the findings of Mascré et al., 2012, yet suggests a slightly higher rate^14^.

The rate of basal cell differentiation may be approximated at steady-state as the rate of epidermal cell shedding. According to our model, up to 30% of the cells in the epidermis basal layer are shed each week^60^.

Further, analysis of clonal dynamics indicated that the majority of Thy1^+^ cellular divisions were asymmetrical, thereby giving rise to one Thy1^+^ SC and one Thy1^−^ progenitor (S → P + S = 94%), while both symmetric self-renewal or differentiation were relatively rare (S → S + S = 3%; S → P + P = 3%) (Fig. 4c). Asymmetrical division was also the main mode of division in the progenitors, giving rise to both a progenitor and differentiated cell (P → P + D = 70%), while the fraction of divisions leading to two progenitors (P → P + P = 12%) or two differentiated cells was lower (P → D + D = 18%) (Fig. 4c).

Next, we constructed a simulation that tracks the fate of individual basal layer cells and quantified the number of divisions that are expected over our experimental timeframe (Fig. 4d, e). We determined that Thy1^+^ SCs divide ~17 times on average, however, a small but finite fraction, ~2.7%, were expected to divide less than 10 times over a 1-year period (Fig. 4f).

As an additional indication of their distinction from other basal layer cells, we performed real time (RT)-PCR analysis on isolated populations of α6^hi^Sca1^hi^Thy1^hi^ SCs and α6^hi^Sca1^hi^Thy1^−^ BCs. As expected, α6^hi^Sca1^hi^Thy1^+^ SCs expressed relatively elevated transcripts of *Krt5, Mt2,* and *Col17a1* (Supplementary Fig. 6a).

Collectively, these results suggest that Thy1 marks a distinct, relatively slow-cycling cell population within the IFE.

In order to functionally validate that Thy1^+^ epidermal SCs indeed behave differently than other basal cells, we sought to evaluate long-term clonal analyses using transgenic mice that target other epidermal cell lineages. However, the widely used lineage-tracing mice available, such as those driven by *Krt14* or *Krt5* promoters, present the issue where Thy1^+^ cells labeling would be enriched as they express significantly higher levels of these basal layer keratins. Thus, we took a different approach and performed long-term clonal analyses using a ubiquitin lineage-tracing mice (*Ubc-Cre*^*ERT2*^*;R26*^*Confetti*^*;* Supplementary Fig. 6b). As expected, following a commonly administered dose of tamoxifen (100 mg/kg) we could detect abundant labeling in the epidermis after 10 days, which presumably included Thy1^+^ keratinocytes (Fig. 4g). Thus, we hypothesized that a very low-dose of tamoxifen (10 mg/kg) could permit random labeling of any basal cell^14^ (Supplementary Fig. 6c). We confirmed that single labeled basal cells could be detected in the epidermis after 4 days post-low-dose induction (Fig. 4h). As an important verification, we employed flow cytometry immediately following low-dose induction (1 day post) to examine whether any Thy1^+^ keratinocytes were initially marked. In this analysis, we determined that only 0.99% of total GFP/YFP^+^ cells comprised the α6^hi^Sca1^hi^Thy1^hi^ keratinocyte population, while 11.5% of α6^hi^Sca1^hi^Thy1^−^ basal cells were marked with GFP/YFP (Supplementary Fig. 6d). Of note, we selectively analyzed GFP/YFP signal due to similar labeling between populations with RFP and CFP at day 1 post induction. Nonetheless, our quantifications indicated a more than 10-fold increase in GFP/YFP-labeled Thy1^−^ basal epidermal cells, attesting to the reliability of this approach to unbiasedly evaluate basal cell retention over time (Supplementary Fig. 6d). Next, we utilized the proliferation dynamics that were determined from lineage tracing of Ubc^+^ basal cells in order to simulate the expected basal clone sizes following random induction by tamoxifen. We found excellent agreement between the model predictions and the measured clone size distributions, thereby corroborating our assessments of clone expansion dynamics (Fig. 4i, j and Supplementary Fig. 6e, f). Finally, we determined a higher fraction of surviving Thy1^+^ basal cell-derived clones over time (displaying basal attachment), in contrast to the Ubc tracing (Supplementary Fig. 6g–i). These data indicate that in contrast to epidermal progenitors, which contribute only superficially to regeneration, Thy1^+^ SCs play a key role in epidermal replenishment over time.

### Thy1^+^ SCs play a key role in epidermal wound repair

**Fig. 5:**
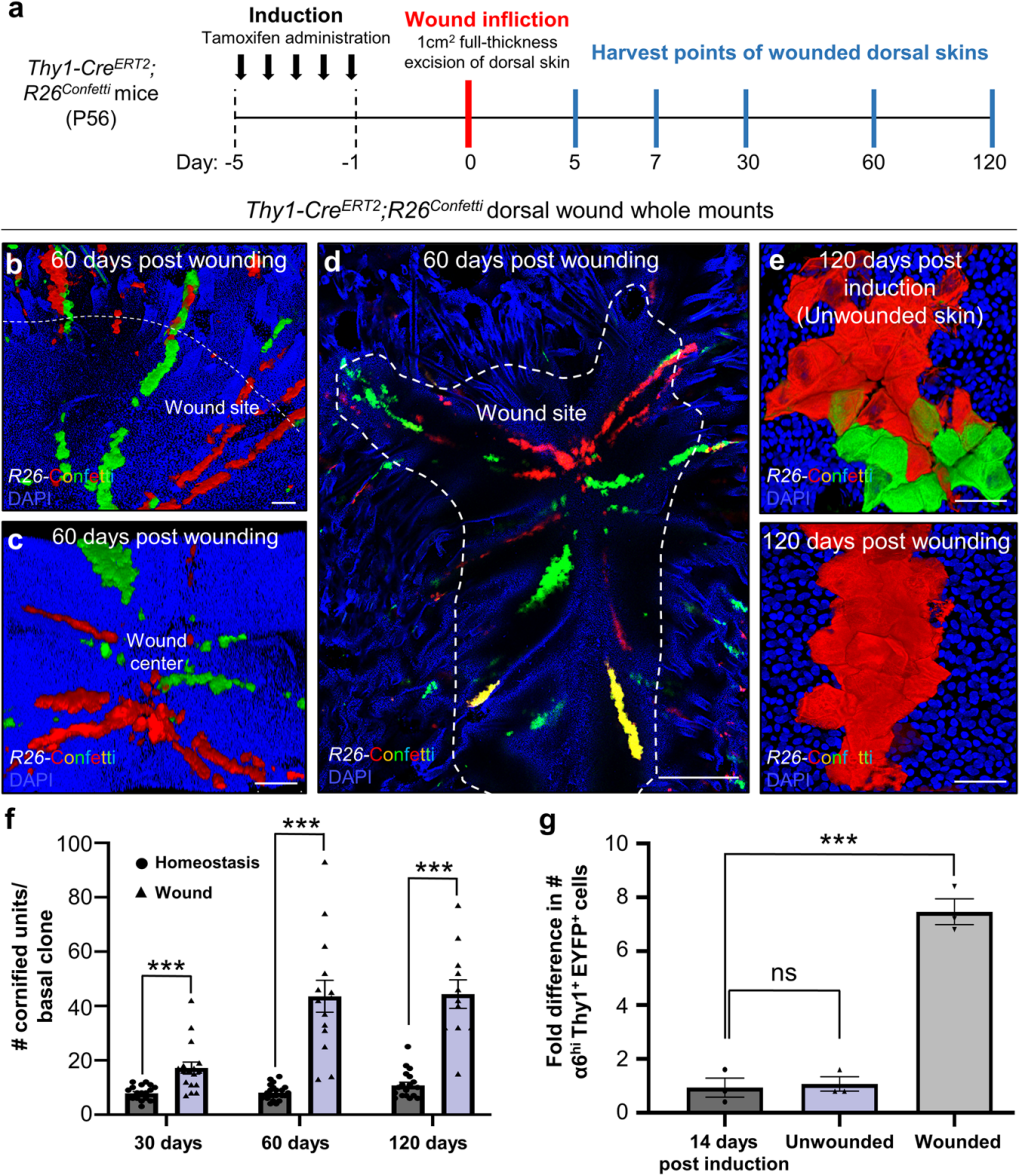
Thy1^+^ stem cells are critical for proper wound repair of the skin. **a** Schematic of induction and wounding. **b**–**d** Thy1^+^ cell progeny migrate to the wound in a clonal fashion and can be detected at day 60 post wound infliction within the wound site [*n* = 3 mice]. **e** At 120 days post wounding, clonal units can be detected in the neo-epidermis that deviate from the regular epidermal proliferative unit (EPU) structure [*n* = 3 mice]. **f** Mean number of cornified units per basal clone at 30, 60, and 120 days post induction in homeostatic (unwounded) skin and wound areas [*n* = 3 mice per time point, 30 clones per mouse]. **g** Fold difference in number of EYFP-labeled α6^hi^Sca1^hi^Thy1^hi^ SCs following a 14-day chase post induction (0 days), and after 14 days in wounded or unwounded dorsal skin [*n* = 3 mice per group]. ns (*P* = 0.778) and ***P *=* 0.0004 (two-tailed unpaired Student’s *t* test). All error bars show ±S.E.M. All source data are provided as a Source Data file. Scale bars: 200 μm (**b**–**d**), 50 μm (**e**).

Since we found that Thy1^+^ SCs contribute to IFE replenishment, we next analyzed their role in the epidermal repair process. Lineage-tracing followed by full-thickness excision wounds (1 cm^2^) was performed on the dorsal skin of adult telogenic (P56) *Thy1-Cre*^*ERT2*^*;R26*^*Confetti*^ mice. Skins were analyzed at 5, 7, 30, 60, and 120 days post wound infliction (Fig. 5a). By 60 days, Thy1^+^ cells and their progeny contributed robustly to wound repair as demonstrated by the presence of fluorescent clones migrating toward the center of the wound (Fig. 5b–d and Supplementary Fig. 7a–f). Labeled cells gave rise to a trail of fully developed polygonal units spanning from the healthy skin towards the wound center (Fig. 5b–d and Supplementary Fig. 7a–c). Notably, in contrast to the unwounded epidermis at 120 post induction, the neo-epidermis displayed an abnormal organization of overlapping fluorescent polygons (Fig. 5e). These results indicate that Thy1^+^ SCs generate trails of persisting clones along their migration path as part of their commitment to restoring the wounded epidermis.

It has been proposed that progenitors are responsible for maintenance of the epidermis while slow-cycling SCs extensively fuel the repair process^12,14^. To examine the regenerative potential of Thy1^+^ SCs upon injury, we examined the epidermal area marked by labeled cells in unwounded and injured lineage-traced *Thy1-Cre*^*ERT2*^*;R26*^*EYFP*^ skins. Our end-point analysis indicates, that during injury, Thy1^+^ SCs contribute significantly more to the repair process when compared to the normal epidermis (Fig. 5f). Of note, we confirmed that labeled nGFP^+^ cells in induced *Thy1-Cre*^*ERT2*^*;R26*^*Confetti*^ mice were positive for Thy1 as well as Krt5 on the wound edges (Supplementary Fig. 8a, b). Moreover, in healed WT dorsal skins at 30 days post wounding we could clearly detect the presence of Krt5^+^ Thy1^+^ SCs throughout the neo-epidermis (Supplementary Fig. 8c).

In light of this, we asked whether Thy1^+^ SCs increase self-renewal to replenish the new SC population in the neo-epidermis. For this aim, tamoxifen was administered for three consecutive days at two weeks prior to the infliction of 1 cm^2^ full-thickness excision wounds on the dorsal skin. We performed flow cytometry of the induced epidermis at an initial time point (14 days post induction), which indicated that ~0.23% of the α6^hi^Sca1^hi^Thy1^+^ population were labeled (Fig. 5g). We further examined Thy1^+^ SC labeling in the wound border and uninjured epidermis at 14 days post wounding. Strikingly, we determined that during wound repair the percentage of YFP^+^ cells from the total α6^hi^Sca1^hi^Thy1^+^ SCs increased eightfold to ~1.85%, in contrast to unwounded skins where the population had barely expanded (~0.26%; Fig. 5g).

These data indicate that Thy1^+^ SCs are activated and self-renew disproportionately more in response to wounding than during homeostasis.

**Fig. 6:**
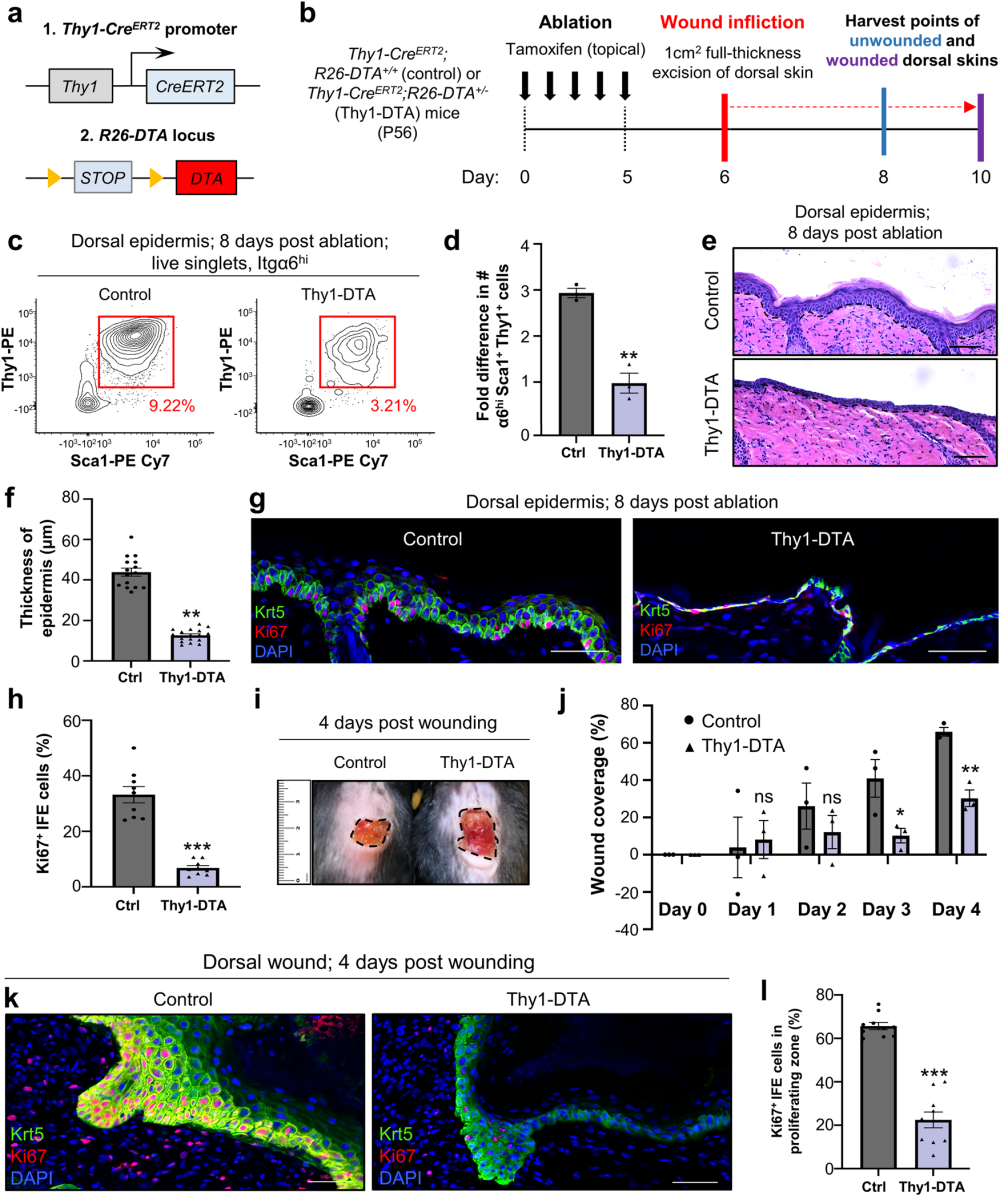
Thy1^+^ cells are required for proper epidermal homeostasis and repair. **a** Genetic strategy used to ablate Thy1^+^ keratinocytes. **b** Schematic of induction, wounding and harvesting of dorsal skins. **c** Representative flow cytometry plots of control and Thy1-DTA dorsal epidermis at 8 days post ablation [*n* *=* 3 mice per group]. **d** Fold difference in numbers of α6^hi^Sca1^+^Thy1^+^ keratinocytes at 8 days post ablation [*n* = 3 mice per group]. **P = 0.0012 (two-tailed unpaired Student’s *t* test). **e** Representative hematoxylin and eosin (H&E) staining of control littermates (*R26-DTA*^*+/+*^) and Thy1-DTA dorsal skins at 8 days post induction [*n* = 3 mice per group]. **f** Quantification of interfollicular epidermal (IFE) thickness after 8 days [*n* = 3 mice per group]. **P *=* 0.006 (two-tailed unpaired Student’s *t* test). **g** Co-immunostaining against keratin-5 (Krt5) and Ki67 in control and Thy1-DTA dorsal skins after 8 days [*n* = 3 mice per group]. **h** Quantification for percentage of Ki67^+^ proliferating cells [*n* = 3 mice per group]. ***P < 0.001 (two-tailed unpaired Student’s *t* test). **i** Representative photograph of control and Thy1-DTA wounds at 4 days post wounding [*n* = 3 mice per group]. **j** Quantification for wound coverage over time [*n* = 3 mice per group]. ns (*P* = 0.836, *P* = 0.411), *P = 0.047, **P = 0.0021 (two-tailed unpaired Student’s *t* test). **k** Wounded dorsal skins immunostained against Krt5 and Ki67 [*n* = 3 mice per group]. **l** Quantification of Ki67^+^ cells in the wound border [*n* = 3 mice per group]. ***P < 0.001 (two-tailed unpaired Student’s *t* test). All error bars show ±S.E.M. All source data are provided as a Source Data file. Scale bars: 50 μm (**e**, **g**, **k**).

Since our lineage-tracing experiments indicated that Thy1^+^ SCs contribute to epidermal homeostasis and skin repair we examined whether they are indispensable for these processes. For this aim, we generated conditional floxed Diphtheria Toxin A (*R26-DTA*) mice crossed to *Thy1-Cre*^*ERT2*^ mice (Fig. 6a; henceforth called Thy1-DTA) for ablation of Thy1^+^ cells, which we compared to *Thy1-Cre*^*ERT2*^ littermate controls that did not inherit the *R26-DTA* allele. Mice were treated with five daily topical applications of tamoxifen and harvested after eight days (Fig. 6b). Ablation of the Thy1^+^ keratinocyte population was confirmed using flow cytometry, which indicated an approximately threefold decrease in α6^+^Sca1^+^Thy1^+^ SCs in contrast to the tamoxifen-treated littermate control (Fig. 6c, d and Supplementary Fig. 9a). It should be noted that we still detected the presence of a large number of homeostatic CD3^+^ DETCs throughout the epidermis post ablation (Supplementary Fig. 9a, b).

Given that our strategy for eliminating a large portion of the Thy1^+^ basal cell population was successful, we next examined whether there were any visible alterations of the epidermis. We first evaluated thickness and morphology of the treated epidermis via Hematoxylin and Eosin (H&E) staining, which revealed altered morphology of different layers within the IFE upon Thy1^+^ cell ablation (Fig. 6e). Specifically, the basal layer keratinocytes appeared to lose their columnar structure and displayed a flattened morphology (Fig. 6e). Furthermore, in contrast to the control epidermis which exhibited a homogenously dense cornified layer, the cornified layer was significantly thinner and inconsistent in skins lacking Thy1^+^ cells (Fig. 6e). We next quantified the thickness of the epidermis and found a nearly three-fold reduction in size, in contrast to the control (Fig. 6f). Of note, we did not detect a significant difference in the thickness of the dermis, indicating that the observed phenotype was predominant in the epidermis (Supplementary Fig. 9b). Given that Thy1^+^ cell ablation resulted in a thinner epidermis we next investigated the effect on the different epidermal layers. Both Krt5 and the differentiation marker Krt10 displayed atypical expression patterns in accordance with the altered morphology of the basal layer (Fig. 6g and Supplementary Fig. 9c). We also examined whether cell proliferation could be an underlying cause for the decrease in epidermal thickness. For this aim, we performed co-immunofluorescence with the Ki67 proliferation marker, which indicated a dramatic decrease in the number of proliferating Ki67^+^ basal cells (Fig. 6g, h). These data attest to the specificity of our approach and suggest that Thy1^+^ SCs play a key role in epidermal homeostasis.

Since Thy1^+^ cell ablation resulted in a strong effect on epidermal homeostasis we next investigated the importance of the Thy1^+^ SC population in wound healing. For this aim, we performed a 1 cm^2^ excision wound on the dorsal skins of induced control littermates and Thy1-DTA mice and monitored the repair dynamics. Our data indicate that ablation of Thy1^+^ SCs leads to significant delays in re-epithelialization throughout the examined time points (Fig. 6i, j). Additionally, we immunostained against Ki67 and, alike the effects in epidermal homeostasis, we observed a significant decrease in cell proliferation adjacent to the wound border (Fig. 6k, l).

**Fig. 7:**
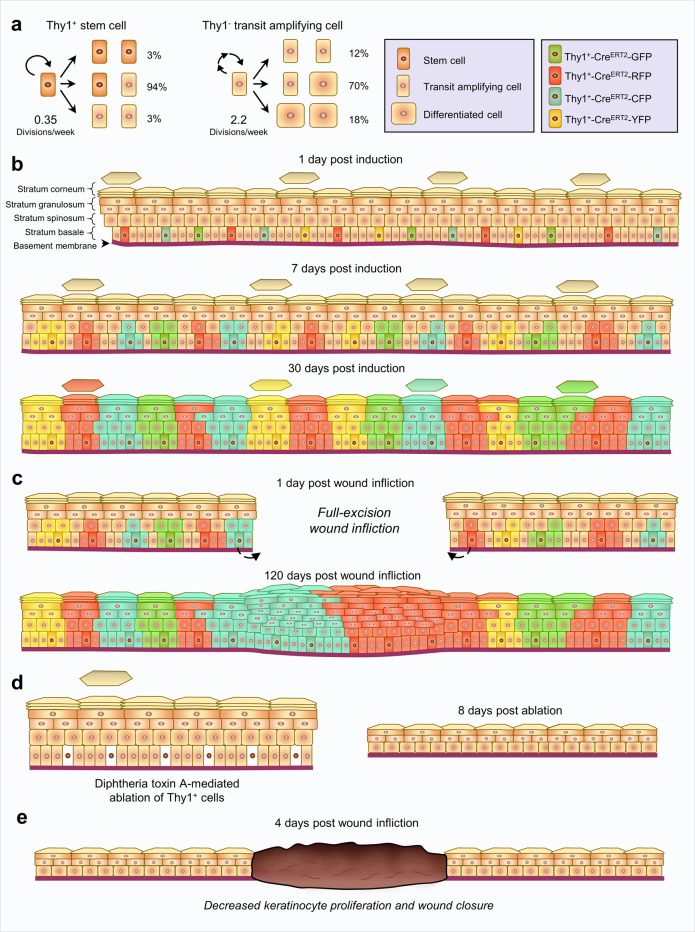
Schematic of Thy1^+^ stem cell dynamics and contributions in the mouse skin. **a** Division fates of Thy1^+^ stem cells and progenitor cells in the mouse epidermis. **b** Clonal Thy1^+^ basal cell-derived events post induction. **c** Clonal Thy1^+^ basal cell-derived events post induction and wounding of the skin. **d**, **e** Outcome of Thy1^+^ cell ablation in the (**d**) homeostatic, and (**e**) wounded mouse skins.

The findings from these ablation experiments indicate that Thy1^+^ SCs play a key role in fueling epidermal homeostasis and driving wound repair in the mouse skin (Fig. 7).

## Discussion

The epidermis consists of distinct compartments, such as the hair follicle (HF) and the interfollicular epidermis (IFE)^3,61^. Under normal conditions, these epidermal compartments are maintained by different populations of stem cells (SCs)^1,62,63^. In contrast to the HF, which has been extensively investigated and shown to house various subpopulations of HFSCs^13,15,16,40–46^, it is still unclear whether the IFE is replenished by a single population of equipotent progenitors or through a hierarchical organization of SCs and progenitors that display distinct proliferative potential^7–9,13–15,17–26^.

In this study, we have identified a distinct population of basal SCs in the mouse IFE, which can be distinguished by Thy1 expression. As an initial indication, we determined that Thy1^+^ basal cells were located in distinct regions of the tail epidermis that were previously reported to contain label-retaining SCs^15,25^.

Probing further, both RNA-seq and immunofluorescence analyses indicated that differential Thy1 expression could resolve two distinct populations, including one that we characterize herein as a Thy1-expressing epidermal SC population and another classical Thy1/CD3/TCR-expressing dendritic epidermal T cell (DETC) population^31^. We suspect that although Thy1 has been described as a marker of SCs in a variety of tissues^28–30,64^, its widely accepted use as a DETC marker could explain why it was largely overlooked in the epidermal SC field.

One important indication that Thy1^+^ cells represent a basal epidermal SC population is their co-expression of high levels of ScaI, Integrins-α6, β4 and β1, and keratins-5, 14 and 15, which have been proposed to mark and play important roles as epidermal SC markers^22,38,54,55^. Importantly, these markers are absent or expressed minimally in the Thy1^+^ T cell population. Further attesting to the clear distinction of Thy1^+^ basal epidermal cells from T cells, we determined the presence of Thy1^+^ keratinocytes in athymic mice. In support, previous studies have noted the existence of Thy1^+^ cells in the epidermis that do not identify as or differentiate into conventional T cells in vivo or in vitro^65,66^. Importantly, the function and activity of these cells remained largely uncharacterized^65,66^.

The epidermal SC is defined by its abilities to self-renew, display greater clonogenic potential than progenitor cells, as well as their slow-cycling proliferative activity^1,3^. By combining different approaches including in vitro assays, lineage tracing, and mathematical modeling of clonal fate we have identified two different keratinocyte populations: (I) a slow-cycling expanding SC population marked by Thy1, and (II) a transient amplifying population that lacks Thy1. We found that Thy1^+^ SCs proliferate at a significantly lower incidence. Notably, both populations utilize asymmetrical division as their main mode of division. This mode of division and cellular turnover is comparable with the elegant findings of Mascré et al., 2012, which defined a Krt14^+^ slow-cycling SC population in the mouse tail skin^14^. It should be noted that this study utilized very low levels of tamoxifen in order to circumvent labeling the entire IFE basal layer. Since Thy1^+^ SCS express significantly high levels of Krt14 it is tempting to speculate that, upon such induction, high numbers of Thy1^+^ SCs were labeled.

Our findings are in line with observations made in various other tissues where a slow-cycling SC population gives rise to actively cycling progenitors^28,67–72^, suggestive of a common strategy for tissue maintenance.

Another defining feature of an SC is its capability to generate differentiated progeny^73^. By performing multicolored lineage tracing we found that Thy1^+^ SCs can replenish the IFE by generating a mosaic of labeled polygonal clones. Early histological studies of the mouse epidermis have proposed that the IFE is organized in distinct columns termed epidermal proliferative units (EPUs)^59^. According to this model each unit is autonomously maintained by a slow-cycling SC that resides in its center and gives rise to a pool of transit-amplifying progenitors with limited proliferative potential^59^. However, lineage-tracing studies have revealed that these EPUs do not exhibit predictable proliferation dynamics or fixed size^7,8,19^. It should be noted that the Thy1^+^ basal cell-derived polygonal units we observed did not span directly from the basal to the cornified layer as proposed by the EPU hypothesis and although the majority of labeled basal cells aligned vertically into its borders, in many instances the basal and suprabasal cells appeared to cross the projected border. These findings could explain why neighboring units displayed a similar fluorescent protein. Our results are consistent with previous studies that examined the spatiotemporal coordination in the mouse ear skin and reported that cells inhabiting the basal and spinous layers have the flexibility to switch to neighboring columns^8,19^.

Having determined that Thy1^+^ cells meet the requirements of a bona fide SC, one key question that then emerged during our investigations was whether Thy1^+^ epidermal SCs were indeed functionally distinct from other basal cells. Moreover, how do our findings reconcile the ongoing debate on clonal dynamics in the epidermis? By performing long-term IFE clonal analyses using *Ubc-Cre*^*ERT2*^*;R26*^*Confetti*^ mice, we could directly compare the contribution of Thy1^+^ SC progeny to other randomly labeled basal cells. By using this particular methodology, we could anticipate the unbiased labeling of basal cells irrespective of protein expression and thereby circumvent potential caveats that can arise from performing genetic inducible fate mapping studies^74^. Importantly, these analyses added further evidence that Thy1^+^ cells represent a distinctly long-lived slow-cycling SC population in the epidermis.

In support of the hierarchical SC model, it has been elegantly shown that two distinct epidermal SC populations, marked by Dlx1 and Slc1a3, exhibit distinct proliferative and differentiation dynamics and function to replenish morphologically different regions in the mouse tail skin^15^. The transcriptomic analyses in our work indicated that Dlx1 and Slc1a3 were not robustly expressed in the Thy1^+^ SC population. It will be interesting to examine how these SC populations co-exist in the tail skin and whether they display functional plasticity or represent subpopulations of Thy1-expressing cells.

In further support for the epidermal SC, pioneering work from the Nishimura group revealed that epidermal cells marked by high levels of Col17a1 largely undergo symmetric division, whereas cells expressing lower Col17a1 levels tend to divide asymetrically^9–18^. It was also elegantly demonstrated that loss of Col17a1 attenuates clonal selection and causes ageing^9–18^. Our analyses of the scRNA-seq dataset by Joost et al. as well as our own transcriptional profiling experiments indicated exclusively high expression of Col17a1 in the Thy1-expressing keratinocyte population^9–18^. Thus, further investigations into the dynamics of Thy1^+^ SCs in aging skin may be an interesting avenue for future research.

Additionally, it has recently been shown that the development of the growing postnatal epidermis is orchestrated by a single uniform population of developmental progenitors^11^. Therefore, it will be important to examine the importance of Thy1^+^ epidermal SCs in distinct developmental stages, as well as the plasticity of various SC populations in the developing, homeostatic, and aged adult epidermis.

In contrast to epidermal homeostasis, where fast-cycling progenitors are the main contributors^14,73^, it has been proposed that SCs play a vital role during the repair process by supplying fresh cells^14,73^. Our lineage-tracing results indicated that, upon injury, Thy1^+^ SCs and their progeny contribute heavily to wound repair, giving rise to a trail of fully developed units spanning from the healthy skin to the wound center. These findings indicate that not only could Thy1^+^ SCs aid in repopulating the wound, but upon migrating into a new niche they could inhabit and repopulate it. Furthermore, the contribution of Thy1^+^ SCs was not transient, since we could observe massive clones at four months post wounding in both unwounded skins and within the neo-epidermis. More importantly, we determined that Thy1-expressing progeny, in contrast to those of the reported epidermal stem cell marker Axin2^13^, contribute disproportionally more to wound healing than to homeostasis. This finding further supports the notion that slow-cycling SCs contribute significantly to the repair process, while progenitors largely maintain homeostatic cell turnover^12,14^.

Our ablation studies further exposed the non-redundant role of Thy1^+^ SCs in the skin. Ablation of Thy1^+^ SCs significantly reduced the thickness of the IFE, affected the columnar structure of basal cells, and altered the morphology of different layers within the IFE. Furthermore, Thy1^+^ cell ablation resulted in a significant delay in re-epithelialization, indicating a vital role of these basal SCs in both tissue homeostasis and repair. Surprisingly, Thy1^+^ depleted skins and wounds displayed a decrease in cell proliferation within the IFE, which could potentially be explained by mitogenic signals released from resident Thy1^+^ SCs. Such a scenario has been observed for various SC populations^75,76^. Future work examining Thy1^+^ basal SCs and identifying the signals they may secrete, as well as the source of the signals that control their activity, could offer significant advances in the fields of skin SC biology and regenerative medicine. Additionally, given the recent indications that epidermal SCs serve as tumor cells of origin^24^, it will interesting to examine the potential contribution of Thy1^+^ basal SCs to tumor formation and maintenance.

Collectively, our results identify a distinct IFE SC population, which plays a critical role in the replenishment and repair of the epidermis.

## Methods

### Mice

C57BL/6 J (WT), B6.Cg-*Foxn1nu*/J (*Foxn1*^*nu*^), B6;SJL-Tg(Thy1-cre/ERT2,-EYFP)VGfng/J (*Thy1-CreERT2*/Slick-H), B6.Cg-*Ndor1 Tg(Ubc-cre/ERT2)1Ejb*/2 J (*Ubc-CreERT2*), B6.129×1-*Gt(ROSA)26Sortm1(EYFP)Cos*/J (*R26*^*EYFP*^) and B6.129P2-*Gt(ROSA)26Sortm1(CAG-Brainbow2.1)Cle*/J (*R26*^*Confetti*^) mice were purchased from The Jackson Laboratory (USA). B6.129P2-*Gt(ROSA)26Sortm1(DTA)Lky*/J (*R26-DTA*) mice were generously donated by Yuval Shaked (Technion, Israel). All experiments comprised at least three randomly assigned 8-week-old (P56) male and female mice per genotype or treatment group. Mice were housed under specific pathogen-free conditions. All animal studies were conducted in accordance with the approval of the institutional ethics by the Pre-Clinical Research Authority (PCRA) of the Technion-Israel Institute of Technology. In all wound repair experiments mice were sedated with isoflurane. Mice were shaved and treated topically with a depilation cream if required. Full-thickness excision wounds (1 cm^2^) were generated on the dorsal skins and monitored for wound coverage in the following days. At the desired time post wounding, mice were euthanized with CO_2_ and the wounded skins were harvested and prepared for whole mounts or sections.

### Lineage-tracing and ablation

For lineage tracing and ablation experiments of Thy1^+^ cells, tamoxifen was dissolved in DMSO (30 mg/ml) and diluted in PBS (1.5 mg/ml) or diluted directly in corn oil (100 mg/kg). 100–200 µl was injected subcutaneously or tamoxifen dissolved in pure ethanol (50 mg/ml) was applied topically on shaved and depilated mouse dorsal skin for five consecutive days. For *Ubc-Cre*^*ERT2*^*;R26*^*Confetti*^ mice, tamoxifen was diluted in corn oil (100 mg/kg for regular dose and 10 mg/kg for low-dose). Mice were sacrificed at specified time points post induction.

### Flow cytometry and cell sorting

In brief, dorsal skins were shaved and harvested. Underlying adipose tissue was removed before incubation in trypsin/EDTA overnight at 4 °C or 1–2 hours at 37 °C. Epidermis and hairs were collected and filtered before sorting. Cells were analyzed and sorted on a BD FACS AriaIIIu, utilizing antibodies against rat anti-mouse/human Integrin-α6/CD49f-PerCP-eFluor710 (eBioscience, #46-0495-82, 1:100), rat anti-mouse Sca1-PE Cy7 (BD Pharmigen, #558162, 1:100), rat anti-mouse CD90.2-PE (BD Pharmingen, #553005, 1:100), rat anti-mouse CD34-FITC (eBioscience, #11-0341-82, 1:100), CD45-APC (Biolegend, #103112, 1:100), CD3-FITC (Biolegend, #100204, 1:100) and γδ-TCR-FITC (Biolegend, #118105, 1:100). Amnis ImageStreamX Mark II flow cytometer or Zeiss LSM880 confocal microscope was used to visualize cells post sorting. Flow cytometry data were acquired using BD FACSDiva Software 7.0 and processed in FCS Express 7 Flow Cytometry Data Analysis or IDEAS programs.

### Cell culture

Sorted keratinocytes were cultured in hair follicle stem cell (HFSC) media on sustaining J2 feeder cells. HFSC medium was prepared using Dulbecco’s modified Eagle medium (DMEM)/F12 3:1 (Biological Industries) containing L-glutamine (Biological Industries; 1:100), penicillin/streptomycin (Biological Industries; 1:100), 10% chelated fetal bovine serum, 5 μg/ml insulin (Sigma I-5500), 5 μg/ml transferrin (Sigma T-2252), 2 × 10 − 12 M T3 (3,3′-triiodo-L-thyronine; Sigma T-2752), 400 ng/ml hydrocortisone (Sigma H0888), cholera toxin 10^−10^ M and 50 μM CaCl_2_ for α6^+^CD34^+^Sca1^−^ cells or 300 mM CaCl_2_ for Thy1^hi^ and Thy1^−^ keratinocytes. For Rhodanile Blue staining, cells were incubated in 1% Rhodamine and 1% Nile Blue for 20 minutes before being washed with water.

### Histology and immunofluorescence

For whole-mount preparation of tail skin, samples were treated with 5 mM EDTA for two hours at 37 °C to separate skin epithelium from dermis and fixed in 4% paraformaldehyde for 1 hour at room temperature. Dorsal skin or wounded skin samples were treated with 5 mM EDTA for two-four hours at 37 °C to separate skin epithelium from the dermis and fixed in 4% formaldehyde for 1 hour at room temperature. For sections, dorsal or wounded skins were embedded in optimal cutting temperature compound (OCT, Scigen), frozen in −80 °C overnight, and cut into 12 μm sections using CM1860 Leica cryostat. Sectioned samples were fixed in 4% paraformaldehyde for 20 minutes at room temperature. Samples were blocked for two hours, before incubation with primary antibodies overnight at 4 °C. Wholemounts or sections were washed at least three times with PBS. Secondary antibodies were incubated for 1 hour at room temperature followed by four washes with PBS.

The following primary antibodies were used: Ki67 (rat, 1:100, eBioscience: Cat. #14-5698-82, Lot # 2196796), CD90.2 (rat, 1:100, BD Bioscience: Cat. #553000, Lot #7086603), CD104 (rat, 1:100, BD Pharmingen: Cat #553745, Lot #8141649), Mcm2 (rabbit, 1:500, Lot #GR3292032-3), Keratin-10 (mouse, 1:100, Abcam: Cat. #Ab9026, Lot #GR306213-22), Keratin-14 (mouse, 1:100, Abcam: Cat. #Ab7800, Lot #GR3204737-4) and Col17a1 (rabbit; 1:100, Abcam: Cat. #184996; Lot #GR3306426-1). Secondary antibody staining was visualized using antibodies conjugated to Alexa Fluors-488, −546 or −633 (all 1:250). All immunostaining and lineage trace images of section and wholemounts were obtained on a Zeiss LSM 880 confocal microscope and analyzed using ZEN 3.0 software (Carl Zeiss). Images were processed and analyzed using ZEN 3.0 software (Carl Zeiss).

### RNA-seq and RT-PCR experiments

Cells were sorted directly into TRIzol-LS (Sigma). Total RNA was isolated and RNA quality was checked by an Agilent 2100 bioanalyzer (Agilent technologies). Small RNA and mRNA libraries preparation followed as manufacturer’s protocols (Illumina Small RNA v1.5 Sample Preparation Kit and Illumina mRNA sequencing Sample Preparation Kit, Illumina). All libraries were sequenced for single-reads, 100 cycles on the Illumina Genome Analyzer IIx (Illumina). RNA-seq reads were aligned with STAR aligner against the mouse genome version mm10 and annotated with featureCounts to the Ensembl mm10 gtf annotation. Downstream analyses were performed in R using the DESeq2, ggplots2, pheatmap, and EnhancedVolcano packages. Analyses of single-cell RNA-seq data (GSE67602)^27^, were performed in R using the Seurat package.

For RT-PCR experiments, cells were sorted directly into TRIzol-LS (Sigma) and were processed for RNA isolation, precipitation, and cDNA synthesis. RT-PCR was performed using the PerfeCTa SYBR Green FastMix (Quanta), where four independent biological and three technical triplicates were used. Average cycle values were normalized to the housekeeping *Rplp0* gene. Forward (f) and reverse (r) primer sequences are as follows:

Krt5 f: 5′-CTCTCACACACACACCTCTAAC-3′

Krt5 r: 5′-GCTGAACTGACCCACACTATC-3′

Mt2 f: 5′-ACCGATCTCTCGTCGATCTT-3′

Mt2 r: 5′-ACTTGTCGGAAGCCTCTTTG-3′

Col17a1 f: 5′-TACGGTGCTGGCTTGTCCTC-3′

Col17a1 r: 5′-ATGTGTCTGCTCAGCTCTCC-3′

Rplp0 f: 5′-GCGACCTGGAAGTCCAACTA-3′

Rplp0 r: 5′-ATCTGCTTGGAGCCCACAT-3′.

### Statistics and Reproducibility

At least three randomly assigned age-matched mice of mixed sex were analyzed in vivo. In all in vitro experiments, at least three independent cultures were analyzed. All experiments were repeated at least twice with similar results. No statistical method was used to predetermine sample size. No data were excluded from the analyses. Measurements were taken from distinct samples. The Investigators were not blinded to allocation during experiments and outcome assessment. Quantitative analyses were performed using two-tailed unpaired Student’s *t* test, where *p < 0.05, **p < 0.01, ***p < 0.001. GO analysis was performed using GOseq and statistical testing was performed using the Wallenius’ distribution. Statistical testing for volcano plots was performed using Fischer’s Exact test.

### Quantitative analysis of proliferation dynamics in the basal layer

We follow refs. ^14,21^ and assume that cell fate dynamics can be described by a Poisson random process, for which the timing between successive events is uncorrelated. Over several rounds of division, any correlations due to synchrony effects will be rapidly erased from the clonal record and could therefore be neglected. We supposed the IFE is maintained by a population of stem (S) and progenitor (P) cells, following a pattern of balanced stochastic cell fate, whilst allowing for a small imbalance between differentiation and proliferation for the P cells. The model dynamics are described by:

1 $$S\mathop{\to }\limits^{{\lambda }_{S}}\left\{\begin{array}{c}{SS}\\ {SP}\\ {PP}\end{array}\right.\,\begin{array}{c}{r}_{S}\\ 1-2{r}_{s}\\ {r}_{S}\end{array}\,P\mathop{\to }\limits^{{\lambda }_{P}}\left\{\begin{array}{c}{PP}\\ {PD}\\ {DD}\end{array}\right.\,\begin{array}{c}{r}_{P}(1-\Delta /2)\\ 1-2{r}_{P}\\ {r}_{P}(1+\Delta /2)\end{array}$$

where D stands for differentiated cells, which are quickly swept away from the basal layer due to their high stratification rates and thus do not play a role in the basal clonal dynamics. The rates, *λ*_*S*_ and *λ*_*P*_ are measured in units of (1/week) and the probability of each specific cell fate is dependent on the imbalance between symmetric and non-symmetric cell fates and the P cells proliferation/differentiation asymmetry, as expressed by the dimensionless parameters, *r*_*S*_, *r*_*P*_, and Δ, respectively.

To predict the clonal dynamics, we use a Monte Carlo approach and acquire 10^5^ simulated clonal outcomes for each choice of *λ*_*S,P*_, *r*_*S,P*_ and Δ starting from a single S cell in the basal layer. For our simulations, we devised a MATLAB implementation of the Gillespie algorithm, where each cell is tracked separately so that the full round of division statistics for S and P cells can be obtained. To find the best fit to the linage tracing data, we perform a scan over feasible ranges of *λ*_*S,P*_, *r*_*S,P*_, and Δ. For each choice of the input parameters, we compare the experimentally determined fraction of clones, of *f*_*n*_(*t*), with size *n* = 1,2,… cells at different time points, *t* = 1,3,5,7,16,30,60,365 days, and the corresponding probability distribution obtained from our simulations, *Q*_*n*_(*t*). To quantify the goodness of fit for our model predictions, we calculated the Kullback-Leibler (KL) divergence^19^ defined by:

2 $${KL}=\mathop{\sum}\limits_{t={day}1,3,\ldots\!\!,365}\;\,\mathop{\sum}\limits_{n}{f}_{n}(t){\log }\left(\frac{{f}_{n}(t)}{{Q}_{n}(t)}\right)$$

The KL divergence is always positive and is equal to 0, only when *f*_*n*_*(t)* and *Q*_*n*_*(t)* are identical. Performing the above procedure, we find that the KL divergence is minimized for the following parameter values:

3 $${\lambda }_{S}=0.35,\,{r}_{S}=0.03\,{\lambda }_{P}=2.2,\,{r}_{P}=0.15,\,\Delta =0.4$$

We now compare our best-fit predictions to the linage tracing data. The clone size distributions at different time points are shown in Fig. 4d, where we plot the distribution of cell divisions for both S and P cells. Note that this number corresponds to the total rounds of division that a surviving clone member has undergone during 52 weeks. For P-type cells, this number includes the rounds of the division of the preceding S-cells. We find that for the optimal parameters in Eq. (3), the probability of a cloned member to undergo 10 or fewer divisions after 52 weeks is 2.68% for S-type cells, while only 0.23% for P-type cells. Overall, these results indicate a good agreement between our model predictions and the Thy1 lineage tracing data.

The rate parameters (3) were used to simulate the clone size distribution in the basal layer following Ubc random basal cell induction. Based on flow cytometry, we estimate ~8.6% of induced cells to be S-type (Fig. 4i), while the rest are P cells. The model predictions for the mean clone size lie well within the SEM of the measurements and performing a two-sided Kolmogorov–Smirnov test between the predicted and measured clone size distributions at day 84 yields a p-value of 0.004. This analysis serves as further corroboration of the hierarchical cell fate dynamics of Eq. (1) and the rate parameters of Eq. (3).

In the absence of cell death, cellular differentiation will ultimately lead to shedding from epidermis, such that at steady state the rate of shedding is given by the overall rate of differentiation. From Eq. (1), we find that the rate of shedding is given by: *P*λ_P_ (1 + *r*_*P*_Δ), corresponding to shedding of ~30% of the basal cell population each week.

### Reporting summary

Further information on research design is available in the Nature Research Reporting Summary linked to this article.

### Supplementary information

Supplementary Information

Reporting Summary

### Source data

Source Data

## Data Availability

The bulk RNA-seq data generated in this study have been deposited in the GEO database under accession code GSE203405. All other related data are available from the corresponding authors upon reasonable request. Source data are provided in this paper.
