# Supplementary material for: Thy1 marks a distinct population of slow-cycling stem cells in the mouse epidermis
Source: Nat Commun. 2022 Aug 8;13:4628. doi: 10.1038/s41467-022-31629-1 (PMC9360001; doi:10.1038/s41467-022-31629-1)
Supplement: Supplementary file 1 — Supplementary Information [file 41467_2022_31629_MOESM1_ESM.pdf]

Supplementary Information

Thy1 Marks a Distinct Population of Slow-Cycling Stem Cells in the Mouse Epidermis

Elle Koren[1, 2, 7, #], Alona Feldman[1, 2, #], Marianna Yusupova[1, 2], Avihay Kadosh[3], Egor Sedov[1, 2], Roi Ankawa[1, 2], Yahav Yosefzon[1, 2], Waseem Nasser[4], Stefanie Gerstberger[5], Liam B. Kimel[3], Noa Prisalec[1, 2], Samara Brown[6], Sam Sharma[6], Travis Gorenc[6], Ruby Shalom-Feuerstein[4], Hermann Steller[6], Tom Shemesh[3 *] and Yaron Fuchs[1, 2 † *]

[1] Laboratory of Stem Cell Biology and Regenerative Medicine, Department of Biology, Technion Israel Institute of Technology, Israel.
[2] Lorry Lokey Interdisciplinary Center for Life Sciences & Engineering, Technion Israel Institute of Technology, Israel.
[3] Laboratory of Biophysics, Department of Biology, Technion Israel Institute of Technology, Israel.
[4] Department of Genetics and Developmental Biology, The Rappaport Faculty of Medicine and Research Institute, Technion - Israel Institute of Technology, Haifa, Israel.
[5] Massachusetts General Hospital, Boston, Massachusetts, USA.
[6] Strang Laboratory of Apoptosis and Cancer Biology, The Rockefeller University, New York, New York 10065, USA.
[7] Present address: Institute for Stem Cell Biology and Regenerative Medicine, Stanford University School of Medicine, CA, USA.

[#] These authors contributed equally

[†] Lead contact

*** Correspondence:** yfuchs@technion.ac.il and tomsh@technion.ac.il

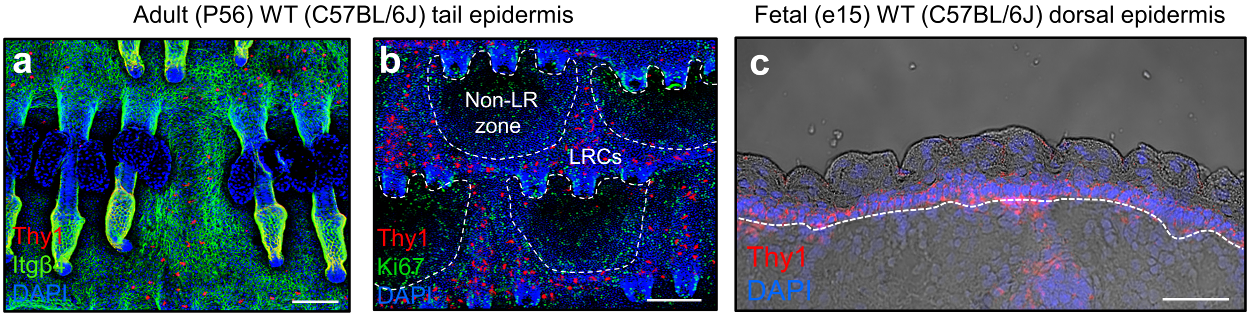

d

Cluster	Basal IFE	Outer bulge	IFE diff.	IFE ker.	Upper HF	Langerhans cells	T cells	SG
Specific markers	*Krt14* *Mt2*	*Postn* *Cd34*	*Krt10* *Ptgs1*	*Lor* *Flg2*	*Krt79* *Krt17*	*Cd207*	*Cd3g* *Thy1*	*Mgst1* *Scd1*

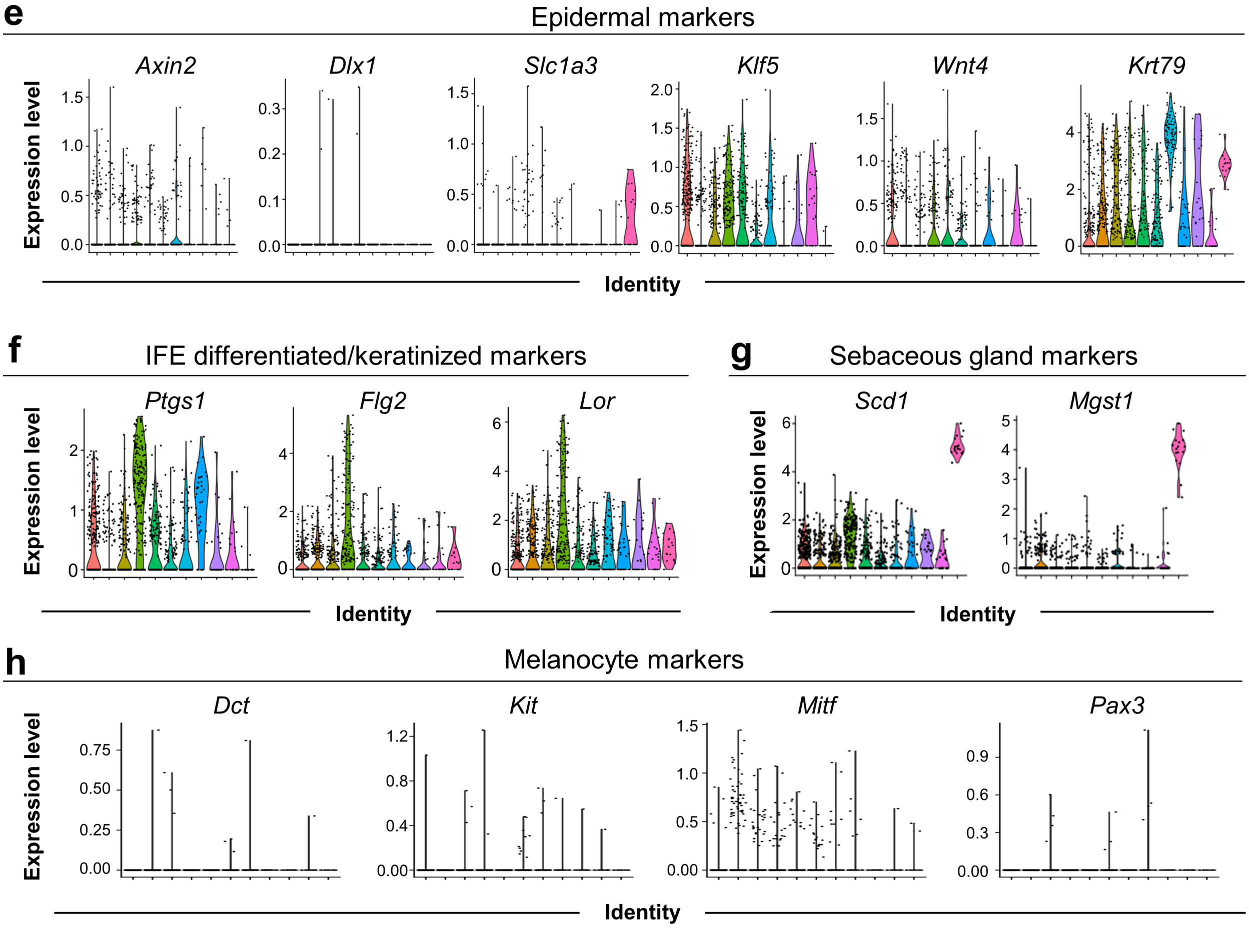

Supplementary Figure 1. Resolution of a transcriptionally distinct Thy1⁺ cell population.

Supplementary Figure 1. Resolution of a transcriptionally distinct Thy1^{+} cell population. (**a**) Tail skin wholemount from adult (P56) wild-type (WT) mouse immunostained against Thy1 and basement membrane marker Integrin-β4 (Itgβ4) [*n* = 3 mice]. (**b**) Exposed label retaining cells (LRCs) and non-label retaining (LR) regions in the interfollicular epidermis (IFE) of tail skin immunostained against Thy1. Ki67 is used as a staining control [*n* = 3 mice]. (**c**) Sectioned dorsal epidermis of embryonic day 15 (e15) WT fetus immunostained against Thy1 [*n* = 3 mice]. (**d**) Markers used for annotation of cell cluster population identities. IFE = interfollicular epidermis; diff. = differentiated; ker. = keratinized; HF = hair follicle; SG = sebaceous gland. (**e**-**h**) Violin plots showing expression level distributions of (e) epidermal, (f) differentiated keratinocyte, (g) sebaceous gland, and (h) melanocyte markers. Dashed white lines demarcate epidermis/dermis boundary unless otherwise indicated. Scale bars: 100 μm (a), 50 μm (c), 20 μm (b).

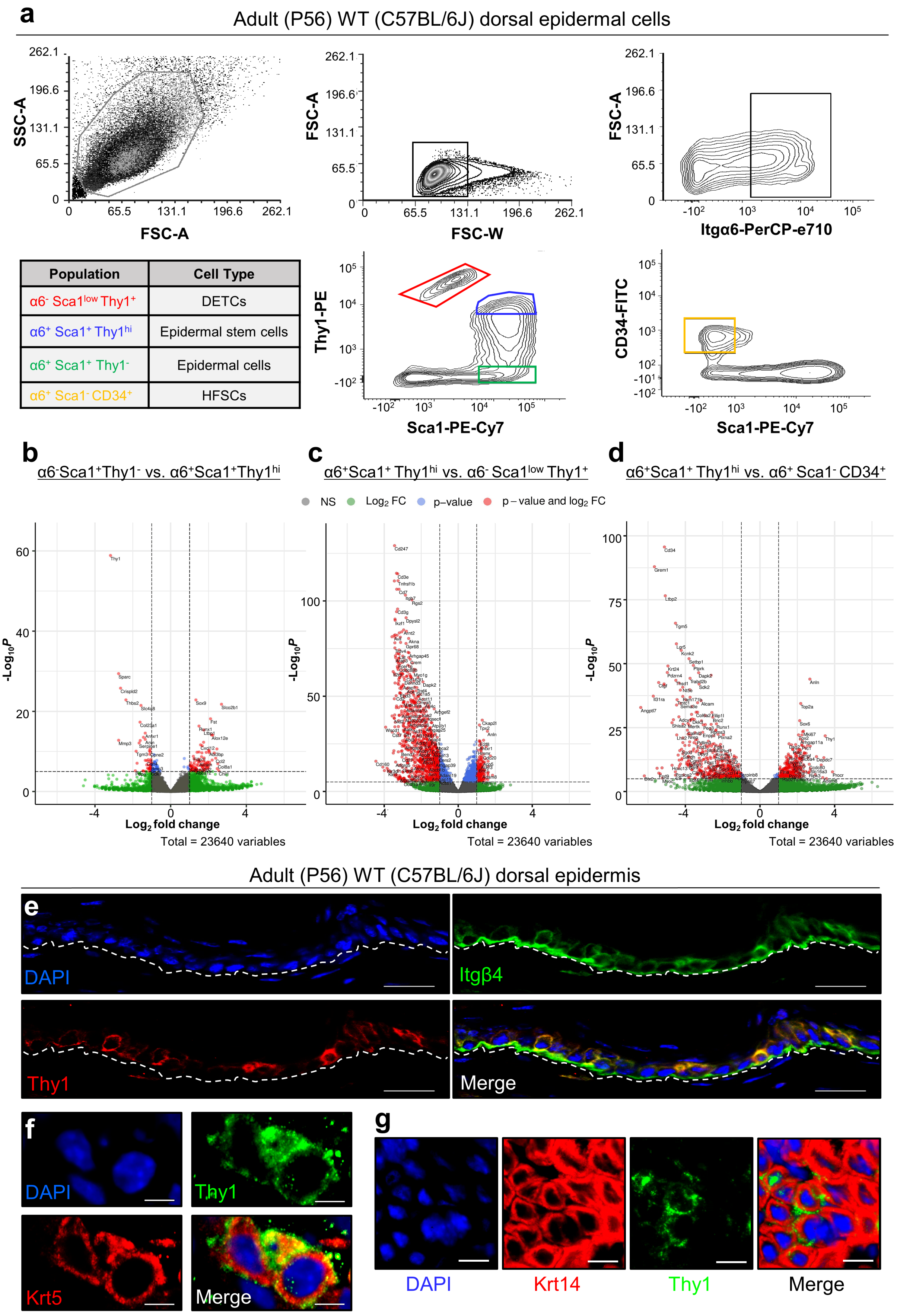

Supplementary Figure 2. Characterization of Thy1$^+$ dorsal keratinocytes.

Supplementary Figure 2. Characterization of Thy1^{+} dorsal keratinocytes. (**a**) FACS strategy for isolating cells used for bulk RNA-seq analyses from WT (C57BL/6J) mice [*n* = 5 pooled mice]. DETC = dendritic epidermal T cell; HFSC = hair follicle stem cell. (**b**-**d**) Volcano plots depicting differentially expressed genes between (b) α6^{-}Sca1^{+}Thy1^{-} vs. α6^{+}Sca1^{+}Thy1^{hi}, (c) α6^{+}Sca1^{+}Thy1^{hi} vs. α6^{-} Sca1^{low} Thy1^{+}, and (d) α6^{+}Sca1^{+}Thy1^{hi} vs. α6^{+}Sca1^{-} CD34^{+} populations [*n* = 5 pooled mice]. Statistical testing was performed using the Fischer's Exact test. (**e-g**) Co-immunostaining of (e) Integrin-β4 (Itgβ4) and Thy1, (f) Krt5 and Thy1, and (g) Krt14 and Thy1 in adult telogenic WT dorsal skins [*n* = 3 mice]. Dashed white lines demarcate epidermis/dermis border. Scale bars: 50 μm (e), 10 μm (g), 5 μm (f).

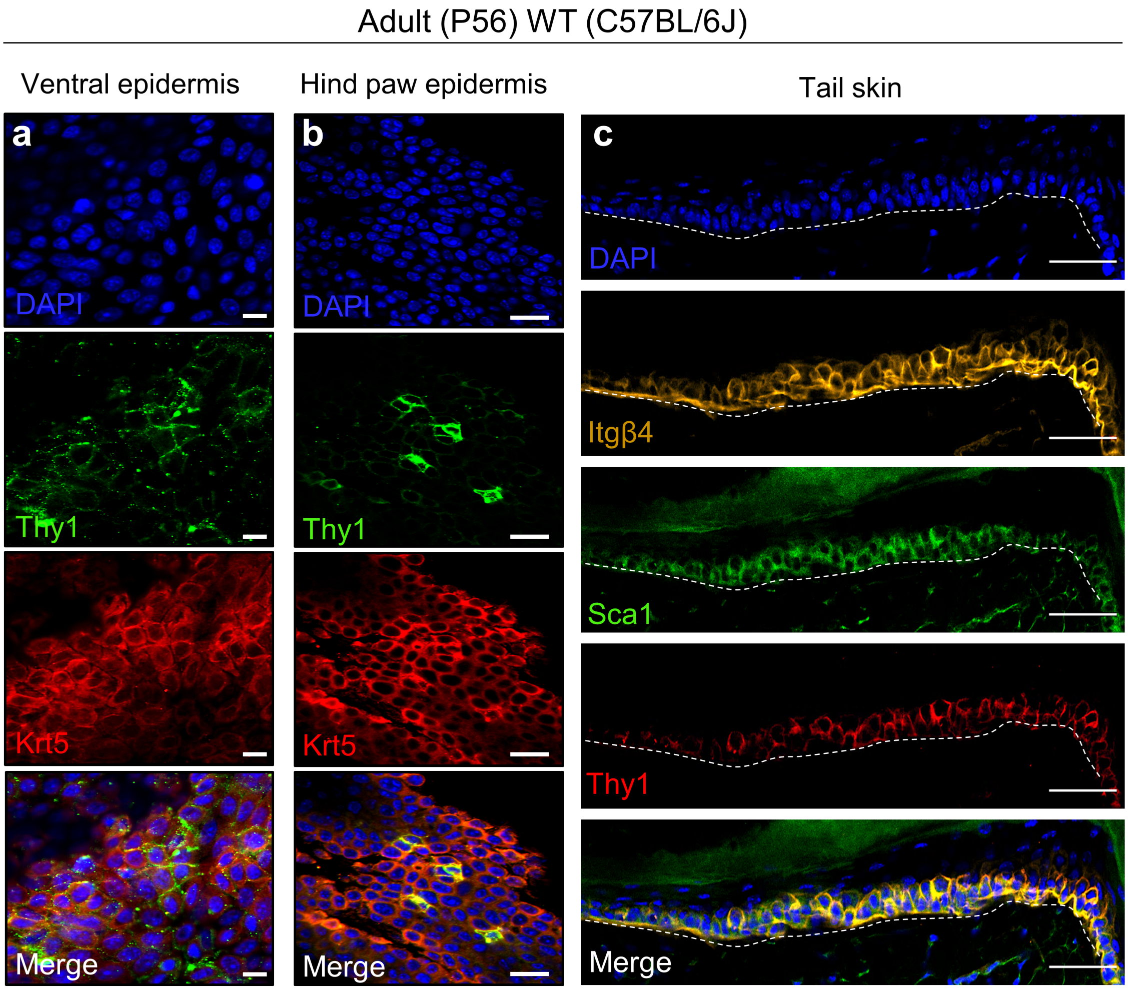

Supplementary Figure 3. Detection of Thy1⁺ keratinocytes in the adult ventral, hind paw and tail skins.

Supplementary Figure 3. Detection of Thy1^{+} keratinocytes in the adult ventral, hind paw and tail skins. (**a, b**) Representative immunostaining against Thy1 and Krt5 in whole mounts of (a) ventral epidermis and (b) hind paw epidermis of adult (P56) WT (C57BL/6J) mice [*n* = 3 mice]. (**c**) Immunostaining against Thy1, Itgβ4 and Sca1 in WT adult (P56) tail skin [*n* = 3 mice]. Dashed white lines demarcate epidermis/dermis boundary. Scale bars: 50 μm (c), 20 μm (b), 10 μm (a).

Adult telogenic *Thy1-Cre^{ERT2};R26^{EYFP}* dorsal epidermis; 1 day

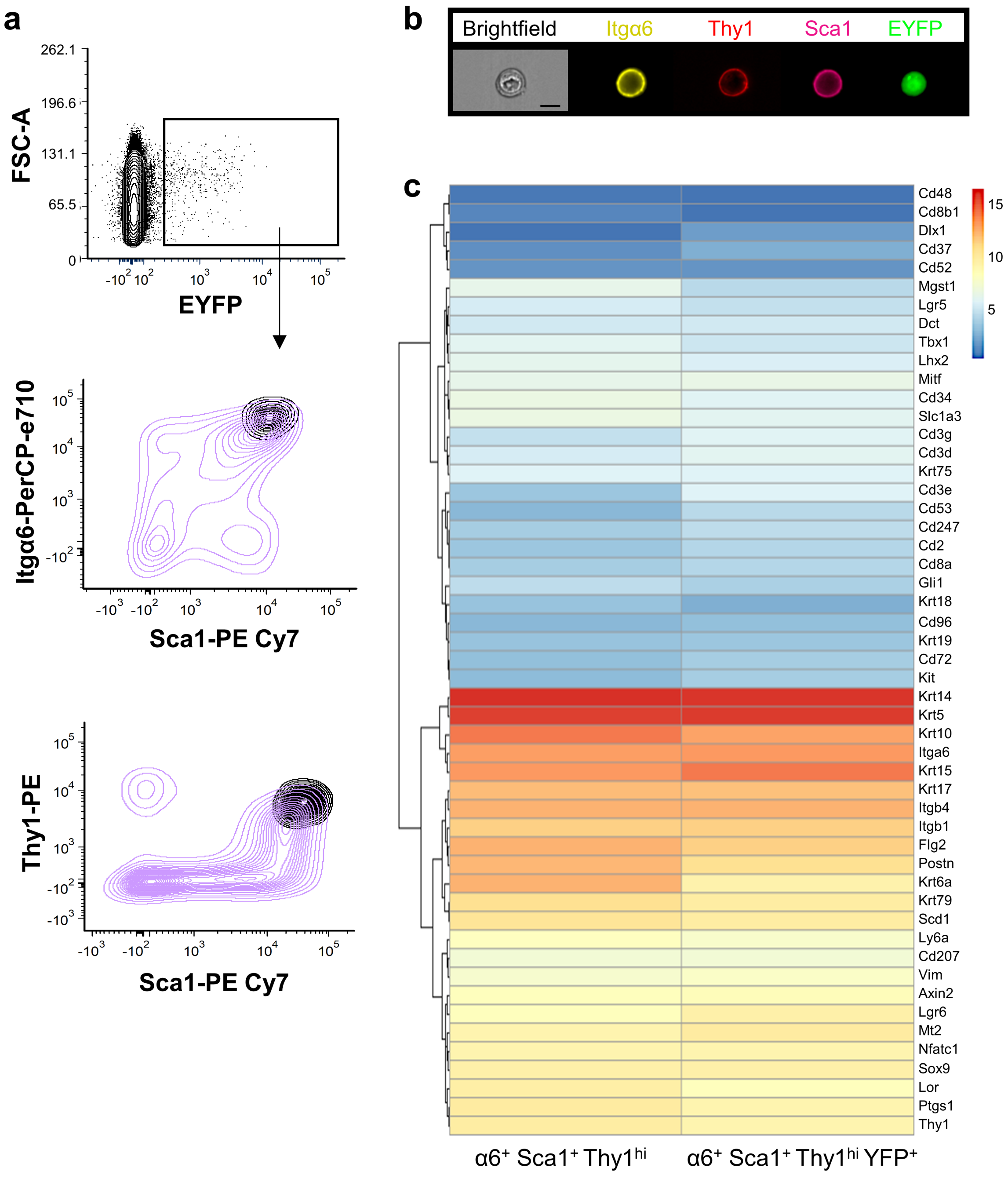

Supplementary Figure 4. Analysis of recombined dorsal epidermal Thy1^{+} cells.

Supplementary Figure 4. Analysis of recombined dorsal epidermal Thy1^{+} cells. (**a**) Flow cytometry of *Thy1-Cre^{ERT2};R26^{EYFP}* dorsal keratinocytes at 1 day post induction gated for EYFP expression from total cells. Gated EYFP^{+} cells are plotted on Itgα6, Sca1 and Thy1 expression [*n* = 5 pooled mice]. (**b**) ImageStream images of sorted α6^{+}Sca1^{+}Thy1^{hi} cell showing EYFP expression [*n* = 10,000 cells]. (**c**) Heatmap of absolute gene expression (RPKM values) based on RNA-seq data comparing sorted populations of (I) α6^{+}Sca1^{+}Thy1^{hi} [*n* = 4 mice], and (II) α6^{+}Sca1^{+}Thy1^{hi}YFP^{+} cells [*n* = 3 mice].

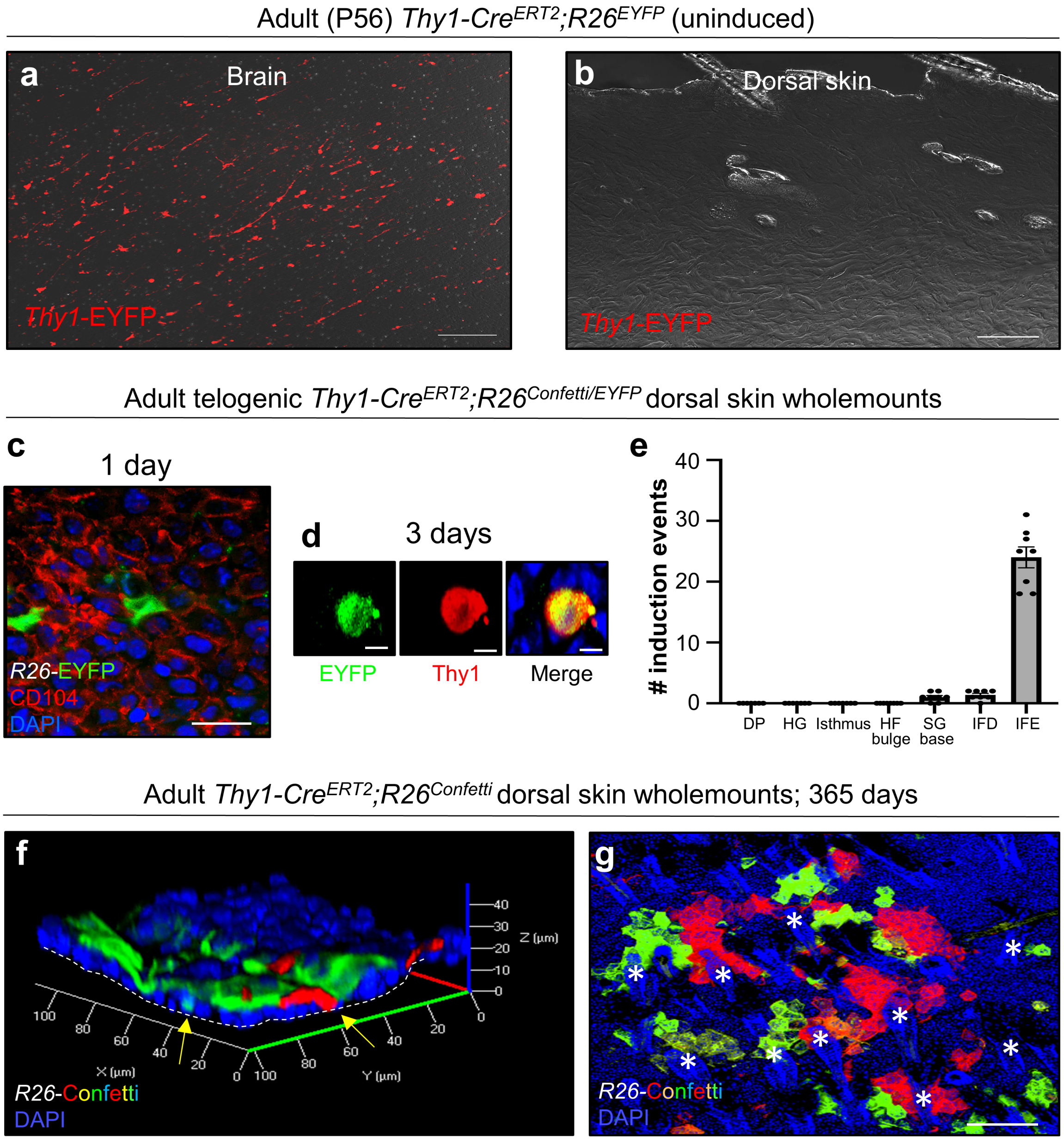

Supplementary Figure 5. Lineage tracing of basal epidermal Thy1⁺ cells.

Supplementary Figure 5. Lineage tracing of basal epidermal Thy1$^+$ cells. (**a**) Constitutive EYFP expression is detectable in neurons within uninduced *Thy1.-Cre^{ERT2};R26^{EYFP}* adult (P56) mouse brain [*n* = 3 mice]. (**b**) Constitutive Thy1$^+$ cell labelling is undetectable in uninduced adult (P56) dorsal skin sections [n = 3 mice]. (**c**) Maximum projection image of *Thy1-Cre^{ERT2};R26^{EYFP}* dorsal skin at 1 day post induction [n = 3 mice]. (**d**) Immunostaining of Thy1 in *Thy1-Cre^{ERT2};R26^{EYFP}* dorsal skins at 3 days post induction [*n* = 5 mice]. (**e**) Mean number of events of label initiation per area at 3 days post induction, including dermal papilla (DP), hair germ (HG), isthmus, hair follicle (HF) bulge, sebaceous gland (SG) base, infundibulum (IFD) and interfollicular epidermis (IFE) [*n* = 5 mice]. Error bars show ±S.E.M. (**f, g**) Confocal images of Thy1 lineage traced dorsal skin at 365 days post induction displaying basal attachment after 1 year (e) and unlabelled intact hair follicles (f) in the IFE [*n* = 4 mice per time point]. Yellow arrows in panel d indicate labelled basal cells. Asterisks in panel e denote HFs. Scale bars: 100 μm (g), 50 μm (a, b), 10 μm (c), 5 μm (d).

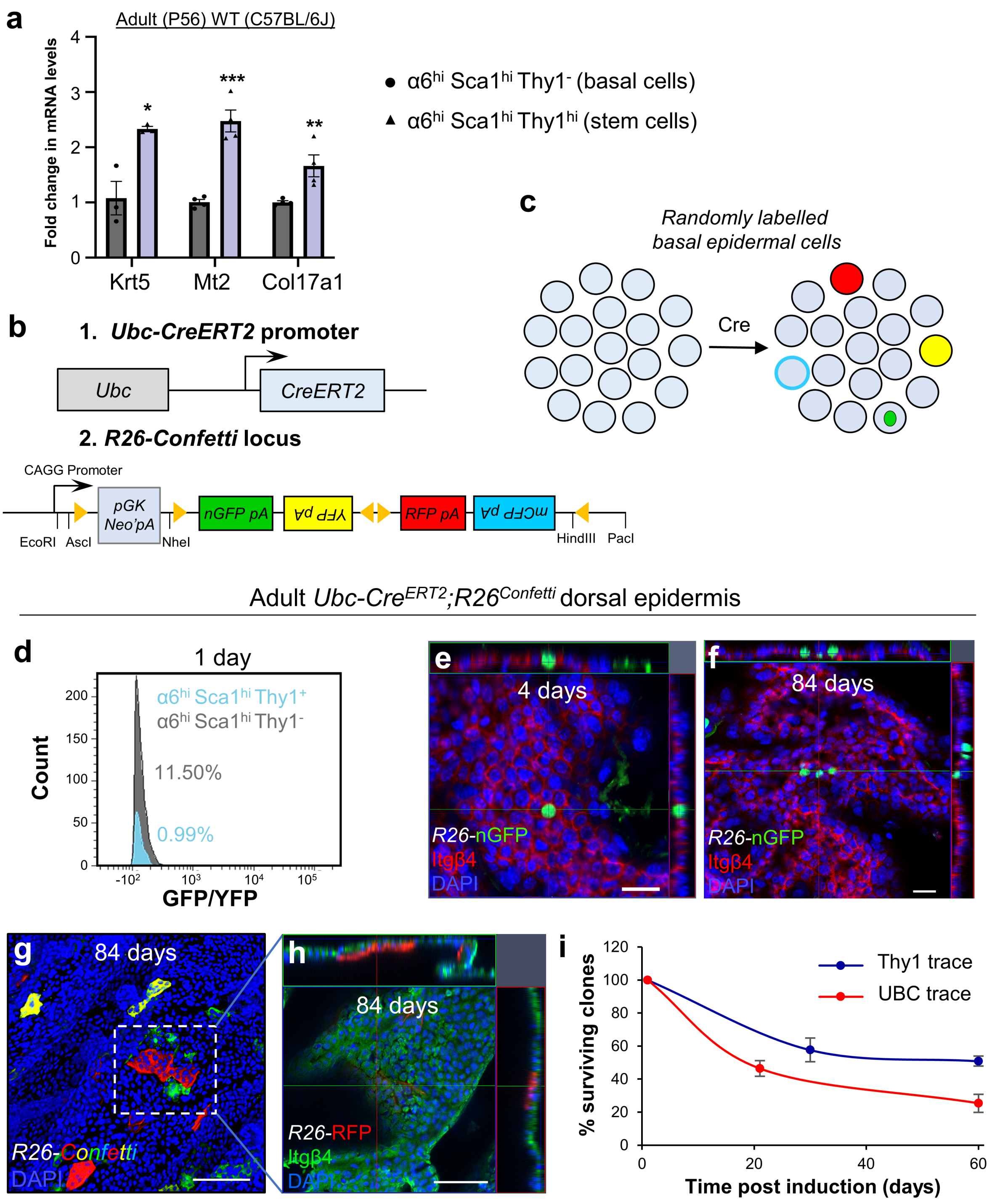

Supplementary Figure 6. Lineage tracing of *Ubc-Cre^{ERT2};R26^{Confetti}* dorsal skins.

Supplementary Figure 6. Lineage tracing of *Ubc-Cre^{ERT2};R26^{Confetti}* dorsal skins. (**a**) Real time (RT)-PCR analysis for relative *Krt5, Mt2* and *Col17a1* transcript levels in sorted populations of α6^{hi}Sca1^{hi}Thy1^{+} stem cells (SCs) vs. α6^{hi}Sca1^{hi}Thy1^{-} basal cells (BCs) [*n* = 4 mice analyzed in triplicates]. Gene expression is normalized to Rplp0. Krt5: **P*=0.03, Mt2: ****P*<0.001, Col17a1: ***P*=0.0062. (**b**) Genetic strategy used to induce multicolor Confetti expression in Ubc-expressing cells. (**c**) Schematic for random labelling of basal cells following low dose of tamoxifen. (**d**) Flow cytometry of *Ubc-Cre*^{ERT2}*;R26^{Confetti}* dorsal epidermal cells at 1 day post induction. Flow plots show α6^{hi}Sca1^{hi}Thy1^{-} and α6^{hi}Sca1^{hi}Thy1^{+} populations positive for GFP/YFP [*n* = 4 pooled mice]. (**e**, **f**) Confocal z-stack images of dorsal skins from Ubc lineage tracing mice harvested at (d) 1 and (e) 84 days post induction [*n* = 3 mice]. (**g, h**) Representative images of Ubc lineage traced dorsal skins at 84 days post induction displaying (h) epidermal clones that lack basal attachment [*n* = 3 mice]. (**i**) Percentages of surviving clones (clones that retain at least one basal cell) in *Thy1-Cre*^{ERT2}*;R26^{Confetti}* and *Ubc-Cre*^{ERT2}*;R26^{Confetti}* dorsal skins at different time points following tamoxifen application [*n* = 3 mice per time point]. All error bars show ±S.E.M. Scale bars: 50 μm (g, h), 20 μm (e, f).

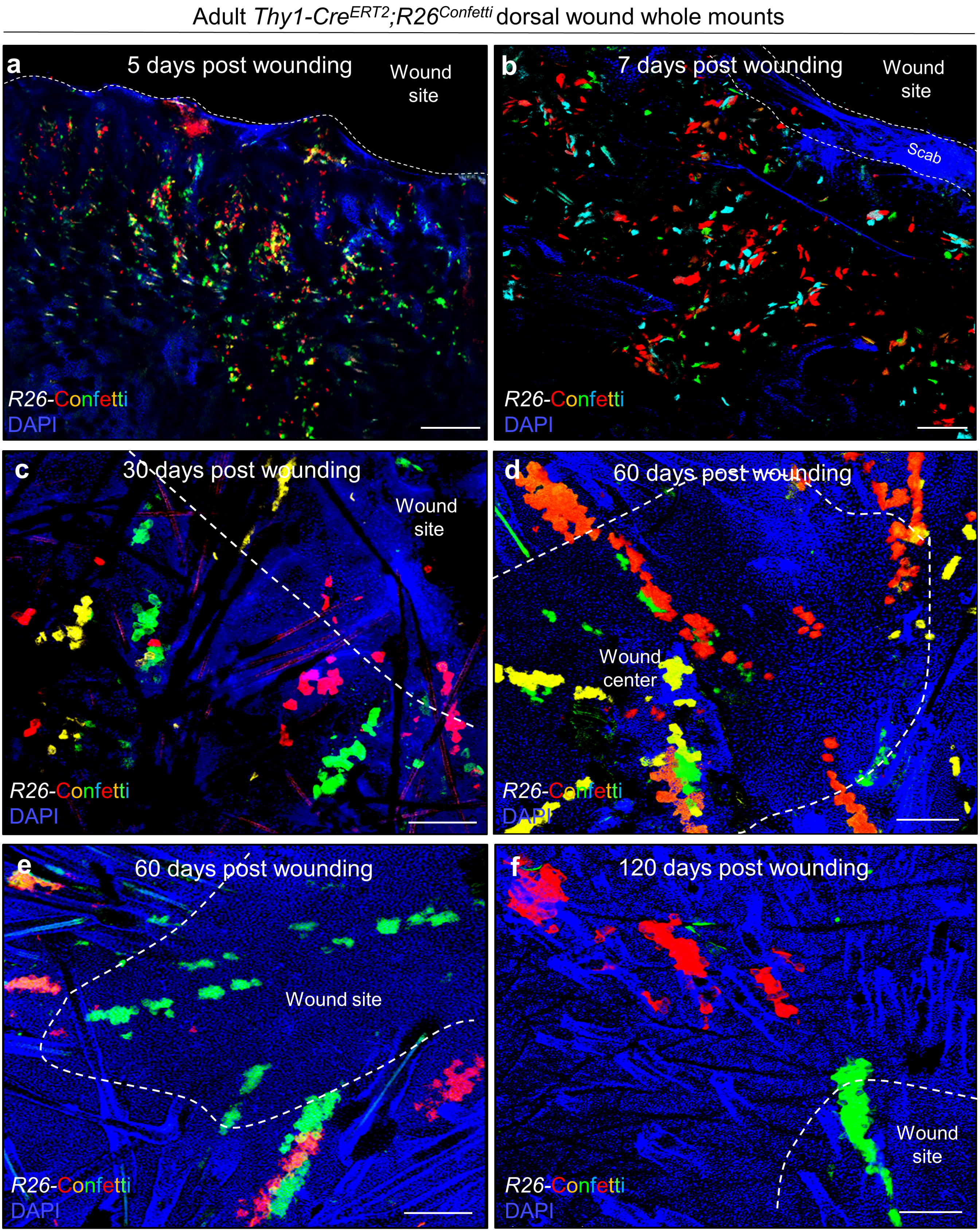

Supplementary Figure 7. Lineage tracing in the *Thy1-Cre^{ERT2};R26^{Confetti}* mouse during wound repair.

Supplementary Figure 7. Lineage tracing in the *Thy1-Cre^{ERT2};R26^{Confetti}* mouse during wound repair. (**a-f**) Representative images of lineage tracing in dorsal skins of *Thy1-Cre^{ERT2};R26^{Confetti}* mice at increasing time points post wounding [n = 3 mice per time point]. Dashed white lines indicate wound site. Scale bars: 200 µm (a-f).

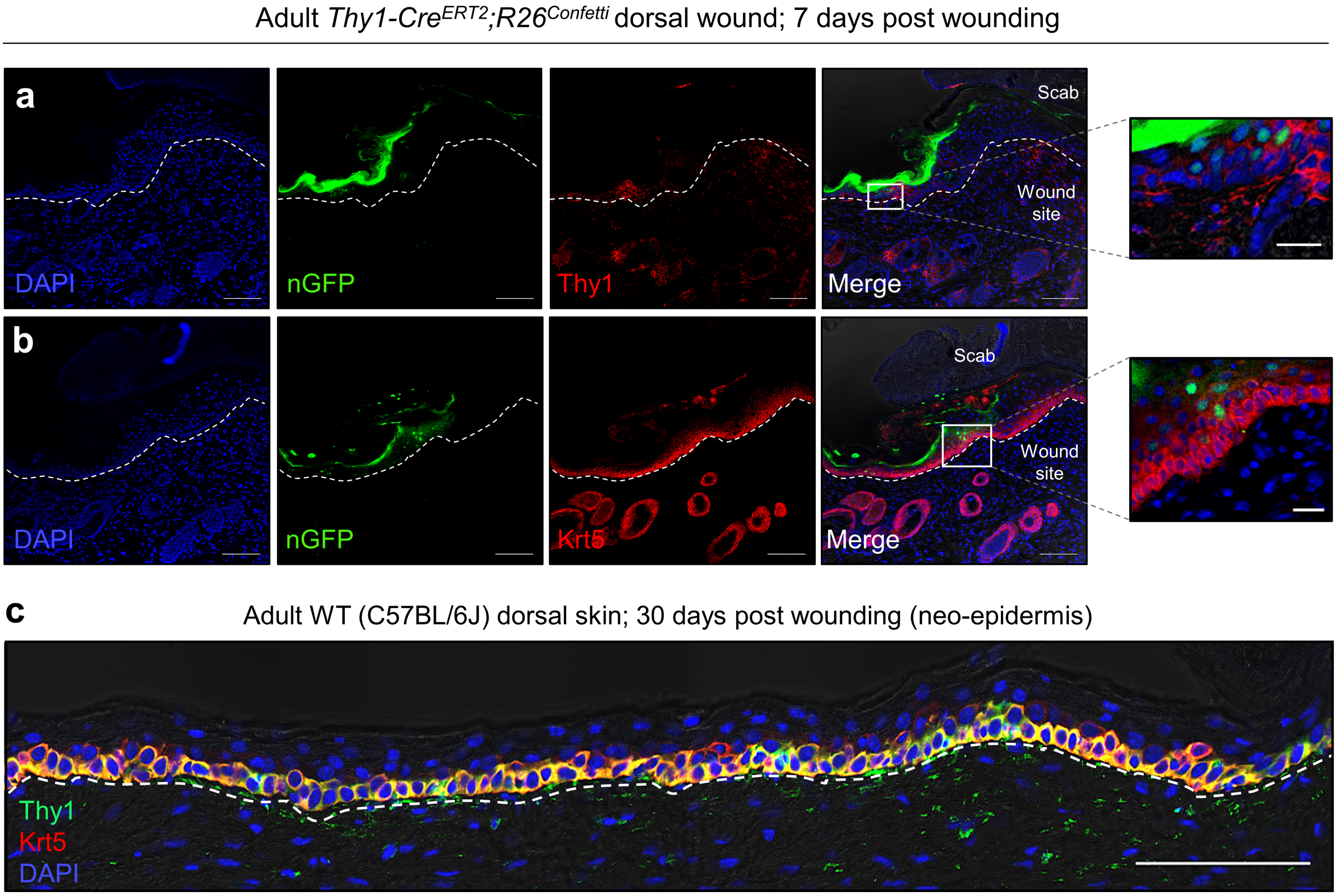

Supplementary Figure 8. Dorsal Thy1⁺ keratinocytes give rise to keratinocytes during wound repair.

Supplementary Figure 8. Dorsal Thy1$^+$ keratinocytes give rise to keratinocytes during wound repair. (**a**) Representative immunostaining against Thy1 in Thy1 lineage tracing mice at 7 days post wounding [*n* = 3 mice]. Inset shows zoom-in of nGFP$^+$Thy1$^+$ cells (white arrowheads) adjacent to the wound site. (**b**) Immunofluorescence against Krt5 in Thy1-traced mice at day 7 post wounding [*n* = 3 mice]. Inset shows zoom-in of labelled nGFP$^+$Krt5$^+$ cells. (**c**) Immunostaining against Krt5 and Thy1 in the healed neo-epidermis at 30 days post wounding [*n* = 3 mice]. Dashed white lines demarcate epidermis/dermis boundary. Scale bars: 100 μm (a, b), 50 μm (c), 20 μm (a inset, b inset).

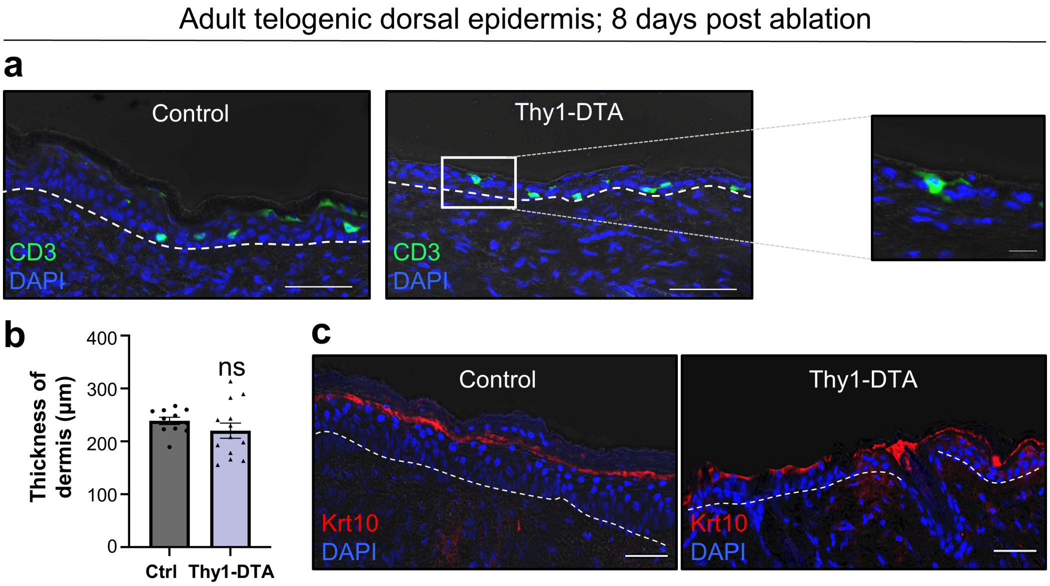

Supplementary Figure 9. Ablation of Thy1⁺ cells does not affect epidermal T cells or the dermis.

Supplementary Figure 9. Ablation of Thy1^{+} cells does not affect epidermal T cells or the dermis. (**a**) Representative immunostaining against CD3 in littermate *Thy1-Cre^{ERT2};R26-DTA$^{+/+}$* (control) and *Thy1-Cre^{ERT2};R26-DTA$^{+/-}$* (Thy1-DTA) dorsal skins [*n* = 3 mice per group]. Insets show zoom-in on homeostatic dendritic CD3^{+} T cell after ablation. (**b**) Quantification for thickness of the dermis in control and Thy1-DTA mice after 8 days induction [*n* = 3 mice per group]. Error bars show ±S.E.M, where ns (no significance) is *P*=0.097 (two-tailed unpaired Student's t-test). (**c**) Representative immunostaining against Krt10 in dorsal epidermis of control and Thy1-DTA mice [*n* = 3 mice per group]. Dashed white lines demarcate epidermis/dermis boundary. Scale bars: 50 μm (a, c), 10 μm (a inset).
