# Supplementary material for: Thy1 marks a distinct population of slow-cycling stem cells in the mouse epidermis
Source: Nat Commun. 2022 Aug 8;13:4628. doi: 10.1038/s41467-022-31629-1 (PMC9360001; doi:10.1038/s41467-022-31629-1)
Supplement: Supplementary file 2 — Reporting Summary [file 41467_2022_31629_MOESM2_ESM.pdf]

nature portfolio

Corresponding author(s): Tom Shemesh, Yaron Fuchs

Last updated by author(s): May 7, 2022

Reporting Summary

Nature Portfolio wishes to improve the reproducibility of the work that we publish. This form provides structure for consistency and transparency in reporting. For further information on Nature Portfolio policies, see our Editorial Policies and the Editorial Policy Checklist.

Statistics

For all statistical analyses, confirm that the following items are present in the figure legend, table legend, main text, or Methods section.

n/a	Confirmed	
☐	☒	The exact sample size (*n*) for each experimental group/condition, given as a discrete number and unit of measurement
☐	☒	A statement on whether measurements were taken from distinct samples or whether the same sample was measured repeatedly
☐	☒	The statistical test(s) used AND whether they are one- or two-sided<br>*Only common tests should be described solely by name; describe more complex techniques in the Methods section.*
☒	☐	A description of all covariates tested
☒	☐	A description of any assumptions or corrections, such as tests of normality and adjustment for multiple comparisons
☐	☒	A full description of the statistical parameters including central tendency (e.g. means) or other basic estimates (e.g. regression coefficient) AND variation (e.g. standard deviation) or associated estimates of uncertainty (e.g. confidence intervals)
☐	☒	For null hypothesis testing, the test statistic (e.g. *F*, *t*, *r*) with confidence intervals, effect sizes, degrees of freedom and *P* value noted<br>*Give P values as exact values whenever suitable.*
☒	☐	For Bayesian analysis, information on the choice of priors and Markov chain Monte Carlo settings
☒	☐	For hierarchical and complex designs, identification of the appropriate level for tests and full reporting of outcomes
☒	☐	Estimates of effect sizes (e.g. Cohen's *d*, Pearson's *r*), indicating how they were calculated

Our web collection on statistics for biologists contains articles on many of the points above.

Software and code

Policy information about availability of computer code

Data collection	ZEN v3.0 (Carl Zeiss), BD FACSDiva v9.0 software, Microsoft Office v16.58
Data analysis	ZEN 3.0 (Carl Zeiss), IDEAS v6.0, FCS Express v7, Image J v1.44, Image Studio v5.2, Microsoft Office v16.58, GraphPad Prism v9.2, STAR aligner (https://github.com/alexdobin/STAR), featureCounts (https://doi.org/10.1093/bioinformatics/btt656), DeSeq2 v1.26.0, ggplots2 v3.3.5, Pheatmap v1.0.12, EnhancedVolcano v1.4.0, Seurat v3.2.2, GOseq 1.38.0.

For manuscripts utilizing custom algorithms or software that are central to the research but not yet described in published literature, software must be made available to editors and reviewers. We strongly encourage code deposition in a community repository (e.g. GitHub). See the Nature Portfolio guidelines for submitting code & software for further information.

Data

Policy information about availability of data

All manuscripts must include a data availability statement. This statement should provide the following information, where applicable:

- Accession codes, unique identifiers, or web links for publicly available datasets
- A description of any restrictions on data availability
- For clinical datasets or third party data, please ensure that the statement adheres to our policy

Source data are provided with this paper. All other related data are available from the corresponding author upon reasonable request.

Field-specific reporting

Please select the one below that is the best fit for your research. If you are not sure, read the appropriate sections before making your selection.

[x] Life sciences [ ] Behavioural & social sciences [ ] Ecological, evolutionary & environmental sciences

For a reference copy of the document with all sections, see nature.com/documents/nr-reporting-summary-flat.pdf

Life sciences study design

All studies must disclose on these points even when the disclosure is negative.

Sample size	No predetermined sample-size calculations were performed. For experiments utilizing mice, a sample size of at least 3 mice (per genotype or treatment) was selected to be consistent with standard practices in biological mouse studies.
Data exclusions	No data were excluded from the analyses.
Replication	All experimental findings are representative of at least three independent biological repeats. To ensure reproducibility, all experiments were repeated at least twice and deemed valid if both rendered similar results.
Randomization	In vitro samples were randomly allocated to different groups/treatments. All mice used for in vivo experiments were sex and age matched.
Blinding	Investigators were not blinded during data collection and analysis.

Reporting for specific materials, systems and methods

We require information from authors about some types of materials, experimental systems and methods used in many studies. Here, indicate whether each material, system or method listed is relevant to your study. If you are not sure if a list item applies to your research, read the appropriate section before selecting a response.

Materials & experimental systems

n/a	Involved in the study	
[ ]	[x]	Antibodies
[x]	[ ]	Eukaryotic cell lines
[x]	[ ]	Palaeontology and archaeology
[ ]	[x]	Animals and other organisms
[x]	[ ]	Human research participants
[x]	[ ]	Clinical data
[x]	[ ]	Dual use research of concern

Methods

n/a	Involved in the study	
[x]	[ ]	ChIP-seq
[ ]	[x]	Flow cytometry
[x]	[ ]	MRI-based neuroimaging

Antibodies

Antibodies used	Conjugated antibodies: rat anti-mouse/human Integrin-α6/CD49f-PerCP-710 (eBioscience, #46-0495-82, 1:100), rat anti-mouse Sca1-PE Cy7 (BD Pharmigen, #558162, 1:100), rat anti-mouse CD90.2-PE (BD Pharmingen, #553005, 1:100), rat anti-mouse CD34-FITC (eBioscience, #11-0341-82, 1:100), CD45-APC (Biolegend, #103112, 1:100), CD3-FITC (Biolegend, #100204, 1:100) and γδ-TCR-FITC (Biolegend, #118105, 1:100).<br>Primary antibodies: Ki67 (rat, 1:100, eBioscience: Cat. #14-5698-82, Lot # 2196796), CD90.2 (rat, 1:100, BD Bioscience: Cat. #553000, Lot #7086603), CD104 (rat, 1:100, BD Pharmingen: Cat #553745, Lot #8141649), Mcm2 (rabbit, 1:500, Lot #GR3292032-3), Keratin-10 (mouse, 1:100, Abcam: Cat. #Ab9026, Lot #GR306213-22), Keratin-14 (mouse, 1:100, Abcam: Cat. #Ab7800, Lot #GR3204737-4) and Col17a1 (rabbit; 1:100, Abcam: Cat. #184996; Lot #GR3306426-1).<br>Secondary antibodies: Alexa Fluors-488, -546 or -633 (all 1:250).
Validation	For all commercial antibodies, we relied on validation statements by manufacturers. All antibody sources and dilutions are provided. Where possible, staining was validated through control tissues (e.g., Thy1 ab on Thy1-deleted tissues).

Animals and other organisms

Policy information about studies involving animals; ARRIVE guidelines recommended for reporting animal research

Laboratory animals	C57BL/6J, B6.Cg-Foxn1nu/J, B6;SJL-Tg(Thy1-cre/ERT2,-EYFP)VGfng/J, B6.Cg-Ndor1 Tg(UBC-cre/ERT2)1Ejb /2J and Rosa26-EYFP/Confetti mice were purchased from Jackson Laboratory (USA). Gt(ROSA)26Sortm1(DTA)Lky mice were received via donation. All experiments comprised randomly assigned eight-week-old male and female mice (across all strains), which were housed under specific pathogen-free conditions.

Wild animals	The study did not involve wild animals.
Field-collected samples	The study did not involve field samples.
Ethics oversight	Approval was granted by the Pre-Clinical Research Authority (PCRA) of the Technion-Israel Institute of Technology.

Note that full information on the approval of the study protocol must also be provided in the manuscript.

Flow Cytometry

Plots

Confirm that:

- [x] The axis labels state the marker and fluorochrome used (e.g. CD4-FITC).
- [x] The axis scales are clearly visible. Include numbers along axes only for bottom left plot of group (a 'group' is an analysis of identical markers).
- [x] All plots are contour plots with outliers or pseudocolor plots.
- [x] A numerical value for number of cells or percentage (with statistics) is provided.

Methodology

Sample preparation	Dorsal skins from eight-week-old mouse were shaved and harvested. Underlying adipose tissue was removed before incubation in trypsin/EDTA overnight at 4°C or 1-2 hours at 37°C. Epidermis and hairs were collected and filtered before staining and sorting.
Instrument	BD FACS AriaIIIu
Software	BD FACSDiva™ Software
Cell population abundance	Cells were sorted by setting a predefined purity mask (16/32). Purity was examined by fluorescence microscopy post sorting (>95%).
Gating strategy	Unstained cell samples were used to set PMT voltages and gating of FSC-A/SSC-A. Dead cells and debris were gated out according to DAPI, FSC and SSC properties. Live singlets were gated using FSC-W vs. FSC-H/DAPI. Histograms and contour plots were used to validate low, medium and high fluorescence levels.

- [x] Tick this box to confirm that a figure exemplifying the gating strategy is provided in the Supplementary Information.
